# TP53INP1 exerts neuroprotection under ageing and Parkinson’s disease-related stress condition

**DOI:** 10.1038/s41419-021-03742-4

**Published:** 2021-05-08

**Authors:** Emilie Dinh, Thomas Rival, Alice Carrier, Noemi Asfogo, Olga Corti, Christophe Melon, Pascal Salin, Sylviane Lortet, Lydia Kerkerian-Le Goff

**Affiliations:** 1 Aix Marseille University, CNRS, IBDM, NeuroMarseille, Marseille, France; 2 Aix Marseille University, CNRS, INSERM, Institut Paoli-Calmettes, CRCM, Marseille, France; 3 Sorbonne Université, Institut du Cerveau - Paris Brain Institute - ICM, Inserm, CNRS, AP-HP, Hôpital de la Pitié-Salpêtrière, Paris, France

**Keywords:** Macroautophagy, Parkinson's disease

## Abstract

TP53INP1 is a stress-induced protein, which acts as a dual positive regulator of transcription and of autophagy and whose deficiency has been linked with cancer and metabolic syndrome. Here, we addressed the unexplored role of TP53INP1 and of its *Drosophila* homolog dDOR in the maintenance of neuronal homeostasis under chronic stress, focusing on dopamine (DA) neurons under normal ageing- and Parkinson’s disease (PD)-related context. *Trp53inp1*^−*/*−^ mice displayed additional loss of DA neurons in the substantia nigra compared to wild-type (WT) mice, both with ageing and in a PD model based on targeted overexpression of α-synuclein. Nigral *Trp53inp1* expression of WT mice was not significantly modified with ageing but was markedly increased in the PD model. *Trp53inp2* expression showed similar evolution and did not differ between WT and *Trp53inp1*^−*/*−^ mice. In *Drosophila*, pan-neuronal *dDOR* overexpression improved survival under paraquat exposure and mitigated the progressive locomotor decline and the loss of DA neurons caused by the human α-synuclein A30P variant. *dDOR* overexpression in DA neurons also rescued the locomotor deficit in flies with RNAi-induced downregulation of *dPINK1* or *dParkin*. Live imaging, confocal and electron microscopy in fat bodies, neurons, and indirect flight muscles showed that *dDOR* acts as a positive regulator of basal autophagy and mitophagy independently of the PINK1-mediated pathway. Analyses in a mammalian cell model confirmed that modulating *TP53INP1* levels does not impact mitochondrial stress-induced PINK1/Parkin-dependent mitophagy. These data provide the first evidence for a neuroprotective role of TP53INP1/dDOR and highlight its involvement in the regulation of autophagy and mitophagy in neurons.

## Introduction

Autophagy dysregulation, inflammation, and oxidative stress are involved in cancer, type 2 diabetes, and age-related neurodegenerative diseases (NDs). Deficient expression of tumor protein 53-induced nuclear protein 1 (TP53INP1) has been identified as a factor linking these mechanisms in cancer and in metabolic syndrome^1^. In contrast, its role in NDs remains unexplored, although *TP53INP1* has been identified as a susceptibility gene for Alzheimer’s disease^2^ shared with type 2 diabetes^3,4^. In vertebrates, *TP53INP1* belongs to a gene family comprising another member, *TP53INP2*, also known as *DOR* (diabetes-and-obesity-regulated), both encoding dual regulators of transcription and macroautophagy^5^ (here referred to as autophagy). In mice, TP53INP1 and TP53INP2 are encoded by *Trp53inp1* and *Trp53inp2*, respectively. Like other invertebrates, *Drosophila* *melanogaster* harbors only one homologous gene, *dDOR*^5^. *TP53INP1* is over-expressed during stress responses in vitro and in vivo in inflamed tissues^6,7^. *TP53INP1* is a p53-target gene, and conversely, the protein TP53INP1 with nuclear localization participates in the positive modulation of p53 transcriptional activity mediating antioxidant-associated tumor suppressor function^1^. Activation of autophagy can induce TP53INP1 translocation from the nucleus to the cytoplasm^5^, where it notably has the p53-independent anti-oxidant function, in part by contributing to mitophagy, an important mitochondrial quality control mechanism that eliminates ROS-producing damaged mitochondria. TP53INP1 has been reported to interact with PTEN-induced putative kinase 1 (PINK1) and with Parkin^8^, two proteins involved in multiple pathways essential for the maintenance of cell homeostasis under stressful conditions, including mitophagy, whose genes are associated with autosomal recessive forms of Parkinson’s disease (PD)^9^. Mutant mice lacking TP53INP1 (*Trp53inp1*^−*/*−^) show exacerbated response to inflammatory insult, increased ROS levels and decreased antioxidant defense, lowered PINK1 and Parkin levels, and increased abundance of dysfunctional mitochondria^8^. These mice are fatter than wild-type (WT) animals and become more rapidly obese and insulin-resistant under a high-fat diet. Most of our knowledge on TP53INP1 function comes from studies in non-neuronal cells. Its role in neurons has been explored in the context of acute insults, in vitro under copper-mediated neurotoxicity^10^ and in vivo in a model of ischemia-reperfusion^11^, in which its upregulation has been linked to its p53-dependent pro-apoptotic action. The possible cytoprotective function of TP53INP1, suggested by its anti-oxidant and autophagy/mitophagy regulatory action, has not been considered in the context of NDs.

The massive and progressive degeneration of the dopamine (DA) neurons in the substantia nigra pars compacta (SNc) is a main pathological hallmark of PD. These neurons are also preferentially vulnerable to degeneration during ageing, which remains the biggest risk factor for developing idiopathic PD^12^. However, the regional patterns of neuron loss associated with ageing and PD are different^13^, pointing to different adaptive responses to cellular stress in these two conditions. Due to their large polarized morphology and high metabolic demands, the function and survival of SNc DA neurons highly depend on healthy mitochondrial function, as well as on efficient degradation and removal of damaged proteins and organelles through the ubiquitin-proteasome system and autophagy^14^. Both degradation systems are key players in neuroprotection and are dysregulated by increased ROS and cytosolic calcium^15,16^. Furthermore, these systems may decline with aging^17–19^ and have been implicated in PD: α-synuclein forms stable aggregates within cells^20^ and genetic risk factors for PD and genes responsible for familial forms are linked to the autophagy-lysosomal pathway^21^, whose impairment leads to the accumulation of dysfunctional organelles, such as mitochondria, and oxidative stress^22,23^. Remarkably, dysfunctional mitochondrial quality control has been highlighted as a central mechanism for DA neurodegeneration in PD^9,24^. SNc DA neurons thus represent a peculiarly interesting model system to investigate the role of TP53INP1 under ageing and NDs-related stressful conditions, in link with autophagy. Here, we addressed this issue, first by examining the consequences of TP53INP1 deficiency in mouse models. We compared DA neuron loss in *Trp53inp1*^−*/*−^ and WT mice at different ages and at different time points of the degenerative process in a PD model based on viral vector-mediated overexpression of the human α-synuclein gene (SNCA). In parallel, we explored the effects of targeted neuronal overexpression of *dDOR* in fly models of PD. In addition, to gain mechanistic insight, we examined the role of *dDOR* and of *TP53INP1* in the regulation of autophagy and mitophagy in *Drosophila* *melanogaster* and in a mammalian cellular model. The results converge to show that TP53INP1/DOR exerts a neuroprotective action, via the regulation of autophagy and mitophagy, in parallel to PINK1/Parkin-mediated pathways.

## Methods and materials

### Studies in mouse models

#### Animals

All mouse experiments were performed using male C57BL/6 mice deficient for TP53INP1 (*Trp53inp1*^−*/*−^) and littermate controls (WT) hosted at the CRCM Luminy (PSEA) animal facility. *Trp53inp1*^−*/*−^ mice were generated as previously described^25^. The mice were kept under specific pathogen-free conditions and maintained on a 12 h light/dark cycle in stable conditions in terms of temperature, humidity, and ventilation. Water and food were offered ad libitum. Welfare and experimental protocols were carried out in accordance with the European Communities Council Directive (2010/63/EU) for the care and use of laboratory animals and were approved by the national and local ethical committees (Comité d’Ethique de Marseille # 14). The project authorization is registered under the number: APAFIS#25200-2020042315133206 v2 delivered by the French Ministry of Higher Education, Research and Innovation. Surgical procedures were carried out by authorized persons and adequate measures were taken to minimize pain and suffering. Animals were 1, 5, or 21 month-old at the start of the experiments. For each genotype, they were randomly distributed in the control and PD groups and at different time points post-surgery. Only animals with a misplaced injection site in the PD model were excluded from the analyses. Cell counts were performed by an investigator blind to the experimental group. The sample size was based on previous experience and similar to studies in the field.

#### Stereotaxic surgery

Five-month-old mice (WT and *Trp53inp1*^−*/*−^) were anesthetized with an intraperitoneal injection of a mixture of ketamine and xylazine (100 and 10 mg/kg, respectively) and mounted on a stereotaxic apparatus (Kopf Instruments). Injections were made with a 10-μl syringe, connected to the injector (30 G) by polyethylene tubing, and controlled by an injection pump at 0.3 μl/min. A group of WT and *Trp53inp1*^−*/*−^ mice received one μl of AAV2-CBA-human-α-synuclein (AAV-αsyn; 1.5 × 10^13^ vg/mL, done by UNC Vector Core and generously given by Michael J. Fox Foundation) into the left SNc (coordinates defined from Bregma with the incisor bar at −1.0 mm: −2.8 mm AP, +1.35 mm ML, −4.35 mm DV, according to the stereotaxic mouse atlas of Paxinos and Franklin^26^). In a set of preliminary experiments, we confirmed the toxicity of AAV2-CBA-eGFP (1 µl of 8.1 × 10^12^ vg/mL, from Michael J. Fox Foundation)^27^, and the absence of neurodegeneration in animals with intranigral injection of the empty vector (AAV null; 1 µl of 10^13^ vg/mL) (not shown). As controls, we then used uninjected 5 month-old animals rather than the uninjected side of the animals receiving AAV- αsyn, since, in some models, the lesion can progress from the injected side to the contralateral one^28,29^. Four subgroups of 6 WT and 6 *Trp53inp1*^−*/*−^ mice were processed in parallel for behavioral testing then for immunohistochemistry, namely controls and AAV-αsyn-injected animals at 15, 30, and 45 days post-injection (dpi).

#### Behavioral tests

Locomotor activity was assessed using the open field test. Mice were placed in a novel open environment consisting of Plexiglas® opaque walls (45 cm×45 cm × 30.5 cm high). Their activity was automatically recorded for 10 min using a force-based actimetry technique (ActiVMeter, Bioseb, France).

The cylinder test has been used to evaluate akinesia-like deficit. Animals were placed in a Plexiglas® cylinder (10 cm diameter) and video recorded for 10 min. The number of forelimb contacts on the cylinder wall while rearing were counted, considering separately the contacts made with either one (ipsilateral or contralateral) or both (double contacts) forelimbs. In this test, double contacts represent the great majority of contacts made by control animals and are reduced in mice with unilateral loss of DA neurons over a threshold triggering akinesia-like deficit of the contralateral paw. Results are then expressed as the percent of double contacts over the total number of counted contacts.

#### Histological staining

Mice under anesthesia were transcardially perfused with 30 ml 4% PFA in PBS 0.1 M, pH 7.2. Brains were dissected out, left 12 h in the same fixative, and then transferred in 30% sucrose (2 × 48 h). Serial frontal cryosections (40 μm) were performed at the level of SNc, from Bregma −2.8 to −3.8 mm according to the atlas of Paxinos and Franklin^26^, and were collected in a cryoprotectant solution. They were distributed in three sets of every three sections, each set comprising eight sections covering the rostrocaudal extent of the structure. A set of sections was used for tyrosine hydroxylase (TH) immunostaining and subsequent counts of TH^+^ neurons, and another one for dual TH and human α-synuclein immunostaining. After rinsing in PBS, permeabilization in 0.1% Triton X-100 solution and blocking in 5% bovine serum albumin (BSA) solution, sections were incubated overnight at room temperature with chicken anti-TH primary antibody (1:1000, Aves Lab #TYH) alone or together with rabbit anti-human α-synuclein (1:2000, Millipore #AB5038P). After rinsing three times in PBS, sections were incubated for 2 h at room temperature with donkey anti-chicken Cy3 secondary antibody (1:500, Jackson ImmunoResearch #703-165-155) alone or together with donkey anti-rabbit Alexa 488 (Life Technologies #A21206). Stained sections were mounted on coverslips with mounting reagent (FluorSave reagent, Calbiochem) and kept away from light until analysis. The last set of sections was processed for Nissl staining using toluidine blue. After rinsing in PBS, sections were mounted on Superfrost™ slides and left to dry overnight. They were then sequentially rehydrated in PBS for 10 min, incubated in toluidine solution at 0.25%, rinsed twice in PBS, left to dry overnight, immersed twice in absolute alcohol then twice in xylene, and coverslipped with DPX mounting medium (Sigma Aldrich).

#### Cell counting

Counts of TH-stained neurons were performed in a set of eight evenly-spaced sections at SNc level per animal. Regional analysis was performed by subdividing the SNc into two regions, anterior and posterior, separated at the Bregma −3.28 mm level (distinguishable by the shape of the SNc split into two parts by the optical tract) and equal in the number of sections (four sections). Sections were viewed using a Zeiss microscope with AxioImager M2 Apotome from the IBDM microscopy platform. Photomicrographs were taken with the ZEN 2 Blue edition Zeiss software. Using ImageJ, SNc was delineated and nuclear profiles of immunostained cells were counted to exclude any bias that could result from changes in cell soma size or shape among experimental conditions. The total number of labeled neurons was estimated for each animal by summing the values obtained in the eight sections analyzed or the four sections per region and multiplying it by a factor of eight to take into account the staining penetration in the depth of the 40 μm section (15 μm as determined with an acquisition in Z-stacks) and the sampling (one out of every three serial sections, cumulated thickness 120 μm). This number was then corrected using Abercrombie’s correction formula *N* = *n*(*t*/*t* + x), in which *N* is the estimated cell number, *n* the number of profiles counted, *t* the section thickness, and *x* the diameter of the profile counted^30^. The same sections and procedures were used to quantify the number of TH^+^ neurons in the ventral tegmental area (VTA). Cell counts in the SNc were also performed on a set of Nissl-stained sections from the same animals using a Leica microscope at ×40 magnification and the Mercator software (Explora Nova). Based on their distinctive cytological features, only neurons were counted^31^. The same procedure and correction as for TH cell counts were used for the cell number estimation in the total SNc or per region. Results are means ± SEM of the values determined from *n* = 6 to 9 animals per experimental condition.

#### RT-qPCR

Mice were sacrificed by decapitation, the brains quickly removed and immediately frozen. In a cryostat, frozen tissue samples of the anteroposterior extension of the SN were collected using a trocar/cannula system (1 mm diameter). Total RNA was extracted from control (5 month-old), aged (21 month-old), and AAV-αsyn injected mice (15 dpi) SNs, using the RNeasy Plus Universal Mini kit (Qiagen) according to the manufacturer’s instructions. RNA concentration was determined with a nanodrop ND-1000 spectrophotometer (Nanodrop Technologies). RNA was stored at −80 °C until use. Total RNA (0.5 mg) was reverse transcribed using Iscript cDNA synthesis kit (Biorad) with random hexamers. RT-qPCRs were carried out in a total volume of 20 µl using 1/100 of the cDNA produced by reverse transcription, SsoAdvanced Universal SYBER green supermix (Biorad), and primer pair of the gene of interest (Eurofins Operon) using Bio-Rad CFX96 cycler with the following cycling parameters: 1 cycle at 95 °C for 30 s (enzyme activation); 40 cycles at 95 °C for 10 s followed by 60 °C for 30 s (denaturation and annealing). Samples were run in triplicate for each gene analyzed. The following gene-specific primers were designed using Universal ProbeLibrary (ProbeFinder version 2.53 for mice, Roche Diagnostics) and used for analyses: *Trp53inp1*: L: gaatataactcaagtggtcccagaat, R: tgccacacagcagtgaatg; *Trp53inp2*: L: gctcatcatcgacctacagga, R: ccagctctcatccatcaagg. Primer pairs were tested for specificity with the melting curve analysis (0.5 °C increments from 55 °C to 95 °C). *Hprt*, the gene coding hypoxanthine phosphoribosyltransferase was used as an endogenous reference gene. Ct values for *Hprt* were not changed among experiments, confirming the validity of *Hprt* as a reference gene. Several no-template controls, which produced no signal, were also included. Relative target gene concentration was calculated using the 2^−ΔΔCt^ method, which uses normalization to endogenous reference gene and normalization to a calibrator sample (here, mean of the control group values). Results are means ± SEM of 5–8 mice per group, except for the AAV null for which *n* = 3.

#### Statistical analysis

All analyses were performed using GraphPad Prism 7 software, and normal distribution checked using Shapiro–Wilk test before statistical comparisons. Two-way analysis of variance (ANOVA) followed by the post-hoc Holm–Sidak test for multiple comparisons was used for the following analyses: estimated numbers of immunostained neurons between SNc regions or between WT and *Trp53inp1*^*−/−*^ mice at different time points during normal ageing or in the progressive PD model, the evolution of the behavioral scores of *Trp53inp1*^*−/−*^ and WT mice in the PD model, *Trp53inp2* expression between WT and *Trp53inp1*^*−/−*^ mice in the ageing and PD conditions. For the evaluation of *Trp53inp1* expression in WT mice in different conditions, an unpaired *t*-test with Welch’s correction or one-way ANOVA followed by the Holm–Sidak test was used. Results are means ± SEM with individual values plotted. The exact *P* values are indicated within each graph and considered statistically significant when *P* < 0.05.

### Studies in *Drosophila* models

#### Culture and stock

GAL4/UAS fly crosses were grown on a cornmeal-agar diet at 29 °C from the egg-laying to the adult stage. *dDOR* mutant and *UAS-dDOR-F* (“*FENLL*”) *and UAS-dDOR-L* (“*Long*”) lines were from A.A. Teleman^32^, *elav-GAL4*^*C155*^ (neuronal driver), *mef2-GAL4* (muscle driver), *CG-GAL4* (fat body driver), *UAS-mCherry::Atg8a*, *UAS-GFP::Atg8a*, *dPINK1*^*B9*^, *UAS-SNCA*^*A30P*^, *UAS-dParkin_IR* (TRiP line HMS01800), *UAS-dPINK1_IR* (TRiP line JF01203), *UAS-Dicer2* and *UAS-mCD8::GFP* were from the Bloomington Drosophila Stock Center (BDSC), *UAS-mtKeima* was from M.J. Clague and A.J. Whitworth^33^, and *TH-GAL4* was from S. Birman. *UAS-mit::dendra2* line stains mitochondrial matrix and is described in^34^. *UAS-dDOR::GFP* line (C-terminal tag) was generated using the *pUASp-GFP-W* gateway vector from T. Murphy and inserted in the fly genome by random P-element transgenesis.

#### Paraquat sensitivity assay

Paraquat sensitivity was analyzed in 2- to 3-day-old adult flies placed in a vial containing a band of paper soaked with a 2% sucrose, 20 mM methyl viologen (Sigma) solution, or only with 2% sucrose for the control experiments. Survival at 25 °C in the dark was monitored. Three vials of ten flies were independently tested for each condition.

#### Assay of locomotion performance

The fly locomotor performance was determined using a negative geotaxis assay. Ten adult flies were placed in a clean plastic column (25 cm tall × internal diameter 1.5 cm) and tapped to the bottom. Each group of flies was submitted to three forced races at 30 s intervals. After 30 s, the flies at the top of the column (*N*_top_) and the flies remaining at the bottom (*N*_bot_) were counted. The locomotor index was defined as (1/2*(10 + *N*_top_ – *N*_bot_)/10). Results are means ± SD of nine races involving 30 flies in total per condition.

#### Immunolabelling and imaging of DA neurons in fly brains

Adult male fly brains (15 days old) were dissected in cold PBS, fixed for 30 min in 4% PFA in PBS, permeabilized 1 h in 0.5% Triton in PBS, saturated 1 h in 0.5% BSA, 0.1% Triton in PBS, and incubated overnight at 4 °C with anti-TH rabbit antibody (Novus Biologicals, NB300-109) at 1/200 in 0.5% BSA, 0.1% Triton in PBS. After 30 min washing in 0.1%, Triton in PBS, fly brains were incubated 2 h at room temperature with donkey anti-rabbit secondary antibody coupled with Alexa 488, washed for 30 min in 0.1% Triton in PBS, and mounted in 90% Glycerol in 20 mM Tris pH 8. We used one layer of double-face adhesive tape in between the glass slide and the coverslip to prevent tissue squashing. Confocal stacks of whole protocerebral anterior medial (PAM) DA neuron clusters were acquired with an LSM880 Zeiss confocal microscope (40X water immersion lens) in order to determine by manual counting the total number of TH-positive neurons. The number of clusters analyzed per genotype was: 15 for *elav-GAL4*, 16 for *elav-GAL4 UAS-SNCA-A30*, 15 for *elav-GAL4 UAS-SNCA-A30P UAS-dDOR-L*. Color coding of Z-depth was generated using the Color Coder ImageJ Plugin.

#### Live imaging of Drosophila tissue

LysoTracker Deep Red, dDOR::GFP, mCherry::Atg8a, GFP::Atg8a, mit::dendra2 were imaged on living tissue (fat bodies from third instar larvae, adult fly brains, and adult dorsal longitudinal indirect flight muscles) that were rapidly dissected in PBS. When a lysosome staining was required, tissues (fat bodies and adult fly brains) were first incubated with LysoTracker Deep Red at 500 nM in PBS for 2 min. Dissected tissues were then mounted in PBS and immediately imaged under a 63X objective with an LSM880 Zeiss confocal microscope equipped with a GaASP detector in order to prevent photobleaching and phototoxicity (laser intensity below 5%). We imaged neuron cell bodies of the cortical area that surrounds the antennal lobes. For analysis of fly brains stained with Atg8::GFP/Lysotracker or mtKeima, the imaged fields were selected in blind based respectively on Lysotracker staining and mtKeima 458 nm signal.

#### Analysis of autophagy and mitophagy

In fat bodies, lysosomal size and density were measured manually on confocal images from third instar larvae of each condition (three samples analyzed per condition). In adult fly brains, LysoTracker-positive and Atg8a::GFP-positive puncta were automatically counted: each channel was submitted to automatic thresholding and the two-color threshold images were analyzed using the ImageJ Com.Det v.0.4.2 plugin (number of brains analyzed: 20 for *elav-GAL4* control, 14 for *elav-GAL4 UAS-dDOR-F*, 8 for *elav-GAL4 UAS-dDOR-L*). To quantify mitophagy in fly brains, ratiometric images were created using the ImageJ RatioPlus plugin by dividing the mtKeima signal detected at 561 nm (mtKeima in lysosomes) by the mtKeima signal detected at 458 nm (mtKeima in the cytoplasm). Then total pixel intensity of the ratiometric images was normalized to the total mitochondrial area measured after automatic image thresholding to obtain a mitophagy index (number of analyzed brains: 15 for *elav-GAL4* control, 9 for *elav-GAL4 dDOR*^−*/*−^, 9 for *elav-GAL4 UAS-dDOR-F*, 7 for *elav-GAL4 UAS-dDOR-L*).

#### Electron microscopy and histology

Adult thoraxes were dissected, the legs, wings, and ventral appendage insertion being carefully removed, and immediately fixed in 2% paraformaldehyde, 2.5% glutaraldehyde, 5 mM CaCl_2_, 0.1 mM Na cacodylate for 24 h at 4 °C. This was followed by 2 h post-fixation in 2.5% glutaraldehyde, 0.8% osmium tetroxide, 0.1 mM Na cacodylate at 4 °C. For histological analysis, semi-thin Epon plastic sections were stained with toluidine blue. For TEM, ultrathin Epon plastic sections were stained with 2% uranyl acetate and lead citrate and were examined with a Tecnai G2 transmission electron microscope. Four thoraces were analyzed for each condition.

#### Statistical analyses

We used the non-parametric Mann–Whitney *U* test. Flies were randomly picked up from a population of more than 50 individuals and came from different crosses generated with different parents. The sample size was determined based on previous experience and was standard in the field. Analysis was not blinded and no animal or sample was excluded.

### Studies in mammalian cells

#### Cell culture and transfection, mammalian expression vectors, siRNAs, treatments

COS7 cells (ATCC) were grown in Dulbecco’s modified Eagle medium (DMEM, Gibco) with 10% fetal bovine serum (FBS), 1% L-glutamine (Invitrogen), and 1% penicillin-streptomycin (Invitrogen) and tested on a monthly basis for mycoplasma contamination (MycoAlert Mycoplasma Detection Kit, Lonza). They were plated in 24-well cell plates, at a density of 4 × 10^4^ per well, on glass coverslips (12 mm diameter, Thermo Scientific) coated with poly-D-lysine (250 µg/ml). For overexpression studies (plasmid DNA only), cells were transfected with 1 µg of plasmid DNA and 1 µl of DMRIE-C (Invitrogen) per well in 150 µl of Opti-MEM I (Gibco), according to the manufacturer’s instructions; after 5 h the medium was changed to DMEM supplemented with 10% FBS. For experiments involving simultaneous use of siRNAs and plasmids, cells were transfected with 1.5 µl of Lipofectamine 2000 (Invitrogen), 10 pmol of siRNA and 0.2 µg of plasmid DNA per well in 600 µl of DMEM supplemented with 10% FBS, and the medium was not changed. The plasmids used for overexpression studies were: empty pcDNA3-HA and pcDNA3 encoding human N-terminally HA-tagged Parkin^35^, pcDNA3 encoding human N-terminally c-myc-tagged TP53INP1 or human C-terminally YFP-tagged TP53INP1 (TP53INP1-YFP)^36^, and pDsRed2-LC3 (kindly provided by Oliver Kepp, Institut Gustave Roussy). Gene downregulation was achieved with: *PINK1* stealth siRNA^37^ (HSS127945, Invitrogen) or *TP53INP1* siRNA (1027417, Qiagen), with AllStars negative control (Qiagen 1027281). For starvation, the cells were incubated for 4 h in Hank’s Balanced Salt Solution (Gibco). Where indicated, the cells were incubated with 10 µM CCCP (Sigma, C2759, 48 h), bafilomycin A1 (Sigma, B1793, 4 h), or their solvent dimethylsulfoxide (DMSO, Sigma, D5879, 0.05%) as a control.

#### Immunocytochemistry and microscopy

Cells were fixed in 4% paraformaldehyde (Sigma, 252549) in PBS and stained with standard procedures. In brief, cells were incubated with the primary antibody in PBS containing 4% BSA and 0.2% Triton X-100 at 4 °C overnight. They were then rinsed twice in PBS, incubated with secondary antibody in PBS with 4% BSA for 45 min at room temperature. After washing three times in PBS, the coverslips were mounted with Vectashield Antifade Mounting Medium (Vector laboratories). The primary and secondary antibodies used were as follows: anti-Parkin (mouse monoclonal, PRK8, MAB5512, Merck Millipore, 1:1000), anti c-myc (mouse monoclonal, clone E19, Sc-40, Santa Cruz, 1:4000), anti MPPB (rabbit polyclonal, PMPCB, 16064-1-AP, Proteintech, 1:400); Alexa Fluor Green 488 goat anti-mouse IgG (H + L) (A11029, Life Technologies, 1:4000) and Cy3 Red goat anti-rabbit IgG (H + L) (A10520, Life Technologies, 1:4000). Z-stack images were acquired with a Nikon A1R-HD25 confocal microscope driven by the NIS-Elements software Nikon Software and a 60 × oil immersion objective and flattened with the ImageJ software. The percentage of cells without mitochondrial staining was quantified manually using a Nikon Ti Eclipse microscope and a 40 × oil immersion objective, with 50 (siRNA conditions with or without plasmids) or 150 (plasmid only conditions) cells scored from each of three independent wells per condition. The results are means ± SEM of three independent experiments. To quantify autophagy, Z-stack projection images were acquired from 25 to 32 cells per condition with a Spinning disk CSU-X1 confocal microscope (Leica) driven by MetaMorph software (100X immersion oil objective, N.A. 1.4), and processed with ImageJ software. Manually-determined threshold masks were created for LC3-DsRed fluorescent stain to select total cell area and area covered by LC3-positive vesicles. Relative LC3-positive area was expressed as the ratio between the area of the cell covered by LC3-positive vesicles and the total cell area. Fold increase in relative LC3-positive area was calculated as the ratio between the relative LC3-positive area in cells treated with bafilomycin A1 in each specific condition and the relative LC3-positive area in non-starved cells not treated with bafilomycin A1 and with endogenous TP53INP1 levels (control). The results are mean ± SEM of one experiment representative of two.

#### RT-qPCR

To evaluate the efficiency of the siRNA-mediated silencing approach for endogenous *TP53INP1* in COS7 cells, total RNA was extracted using RNeasy Microkit (Qiagen), according to the manufacturer’s instructions, and treated with DNAse I (Qiagen) for 20 min at room temperature to prevent genomic DNA contamination. RNA concentrations were determined by spectrophotometry, using Nanodrop 2000c (THERMO Scientific). Complementary DNA was generated from 500 ng RNA with random hexamers and Superscript II reverse transcriptase (THERMO Fisher Scientific). Real-time PCR was performed with the LightCycler 480 qPCR system (Roche) with SYBR green detection and each of three independent primer pairs (in 5′-to 3′ orientation): L1: gccaaactctcagtcttgcc, R1: aagggtgctcagtaggtgac; L2: cataactcctgccctggtct, R2: tgcccgtgagtcttataagca; L3: tcctccaaccaagaaccaga, R3: tcctccaaccaagaaccaga. Relative gene expression levels were calculated using the 2^−ΔΔCt^ method, with GAPDH used as the reference gene for normalization.

#### Statistical analyses

Analyses were performed using GraphPad Prism 7 software. Normal distribution was checked using the D’Agostino Pearson normality test before statistical comparisons. Data were analyzed by two-way ANOVA. When pertinent (Fig. 7B, C), the Tukey post hoc test was used for multiple comparisons. The sample size was standard and determined based on previous experience. Analyses were not blinded and no sample was excluded.

## Results

### TP53INP1 deficiency triggers additional nigral TH neuron loss in aged mice

**Fig. 1:**
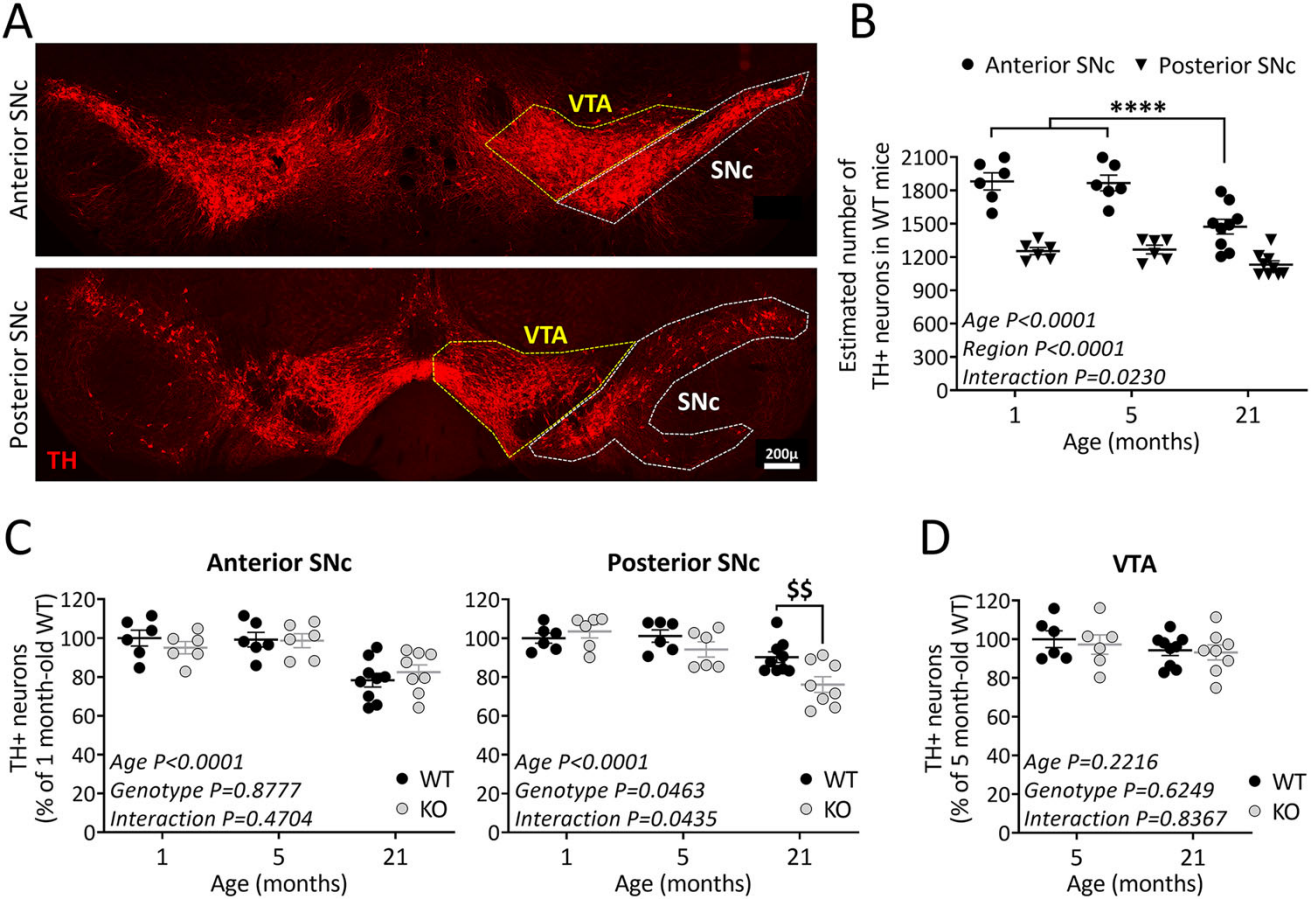
TP53INP1 deficiency impacts the intranigral pattern of ageing-related loss of TH-positive neurons. **A** Representative image of TH immunolabeling in the midbrain. VTA and SNc were delineated for quantification. SNc was subdivided into anterior and posterior parts. Scale bar 200 µm. **B** Effect of ageing on the numbers of TH-positive neurons in the two SNc subdivisions. Estimated total numbers of neurons were compared between 1, 5, and 21 month-old WT mice. Age-related loss of TH+ neurons is restricted to the anterior SNc, which shows higher numbers of these neurons than the posterior part in 1 and 5 month-old mice. ****P < 0.0001 21 versus 1 or 5 month-old mice. **C** Effect of TP53INP1 deficiency on the age-related evolution of TH+ neuron numbers in the anterior and posterior SNc. Counts were performed in WT and *Trp53inp1*^−*/*−^ (KO) mice and expressed as % of 1 month-old WT mice. 21 month-old *Trp53inp1*^−*/*−^ mice show loss of TH + neurons in the anterior SNc equivalent to WT and an additional loss in the posterior SNc. $$*P* = 0.0075 KO versus WT. **D** Effects of ageing and of TP53INP1 deficiency on the numbers of TH+ neurons in the VTA. No significant changes are measured in either condition. In graphs, data are expressed as means ± SEM of n mice per group (1 month-old: 6 WT and 6 KO, 5 month-old: 6 WT and 6 KO, 21 month-old: 9 WT and 8 KO), and results of the two-way ANOVA are indicated. Only significant interaction is symbolized.

To investigate the role of TP53INP1 during ageing, we examined the consequences of its deficiency on the number of TH^+^ neurons in the SNc of juvenile (1 month-old), adult (5 month-old), and old (21 month-old) mice. Based on previous data showing a heterogeneous distribution of DA neurons in the rostrocaudal extent of the SNc^38–40^, we performed a regionalized analysis, subdividing the SNc into anterior and posterior parts (Fig. 1A; see “Methods and materials”). The two-way ANOVA in WT mice showed significant age effect [F(2,36) = 15.9], region effect [F(1,36) = 122], and age × region interaction [F(2,36) = 4.2] (Fig. 1B). Post-hoc comparisons indicated that the numbers of TH^+^ neurons were higher in the anterior versus posterior SNc at all the ages examined, confirming the heterogeneity of the structure. The ageing-related TH^+^ neuron loss was region-dependent: a significant loss was observed in the anterior part but not in the posterior part of the SNc.

We then compared age-related neuronal loss in *Trp53inp1*^−*/*−^ versus WT mice (Fig. 1C). The two-way ANOVA for the anterior SNc showed an age effect [F(2,35) = 16.7] with no genotype effect [F(1,35) = 0.02] and no age × genotype interaction [F(2,35) = 0.8], meaning that TP53INP1 deficiency did not impact the ageing effect in this region. WT and *Trp53inp1*^−*/*−^ mice presented equivalent numbers of TH^+^ neurons at the ages considered and both showed significantly reduced numbers at the age of 21 months versus 1 or 5 months (Fig. 1C). In the posterior SNc, the two-way ANOVA indicated significant effects for age [F(2,35) = 17.7], genotype [F(1,35) = 4.3] and age × genotype interaction [F(2,35) = 3.4], showing that the age-related loss was dependent on the genotype (Fig. 1C). Hence, 21 month-old *Trp53inp1*^−*/*−^ mice showed a significantly reduced number of TH^+^ neurons in the posterior SNc versus WT of the same age, as well as versus 1 or 5 month-old *Trp53inp1*^−*/*−^ mice. Therefore, TP53INP1 deficiency triggered a topographically restricted additional loss of nigral TH neurons with ageing. No significant age- or genotype-related changes in the number of TH^+^ neurons were measured in the VTA, indicating the selective impact of ageing and of TP53INP1 deficiency among mesencephalic DA neurons (Fig. 1D). To verify the ageing-related neuronal loss in SNc, we also performed counts on Nissl-stained sections from 5 and 21 month-old WT and *Trp53inp1*^−*/*−^ mice. In 5 month-old WT mice, the estimated numbers of Nissl-stained neurons were higher than those of TH^+^ neurons, especially for the posterior SNc where DA neurons are intermingled with GABA neurons of the SN pars reticulata [anterior SNc: 2235.9 ± 37.6 (Nissl) versus 1866.4 ± 70.9 (TH); posterior SNc: 2326.5 ± 56.9 (Nissl) versus 1267.6 ± 39.7 (TH)]. Despite these differences, the age and genotype comparisons (supplemental Fig. 1A) confirmed the occurrence of an ageing-related loss of Nissl-stained cells in the anterior SNc that is not affected by TP53INP1 deficiency (two-way ANOVA showing an age effect with no genotype effect and no age × genotype interaction) and the selective impact of TP53INP1 deficiency in the posterior SNc [two-way ANOVA showing a genotype effect only and post-hoc analysis showing a significant difference between *Trp53inp1*^−*/*−^ and WT at the age of 21 months (*P* = 0.0089), but not of 5 months]. Therefore, at least part of the age-related loss of TH neurons in WT and *Trp53inp1*^−*/*−^ mice is attributable to neurodegeneration.

Overall, these results provide evidence for a neuroprotective role of TP53INP1 in male mice under ageing-related conditions and for differential region effects among nigral DA neurons.

### TP53INP1 deficiency exacerbates motor deficits and nigral dopaminergic neurodegeneration elicited by targeted α-synuclein overexpression in mice

**Fig. 2:**
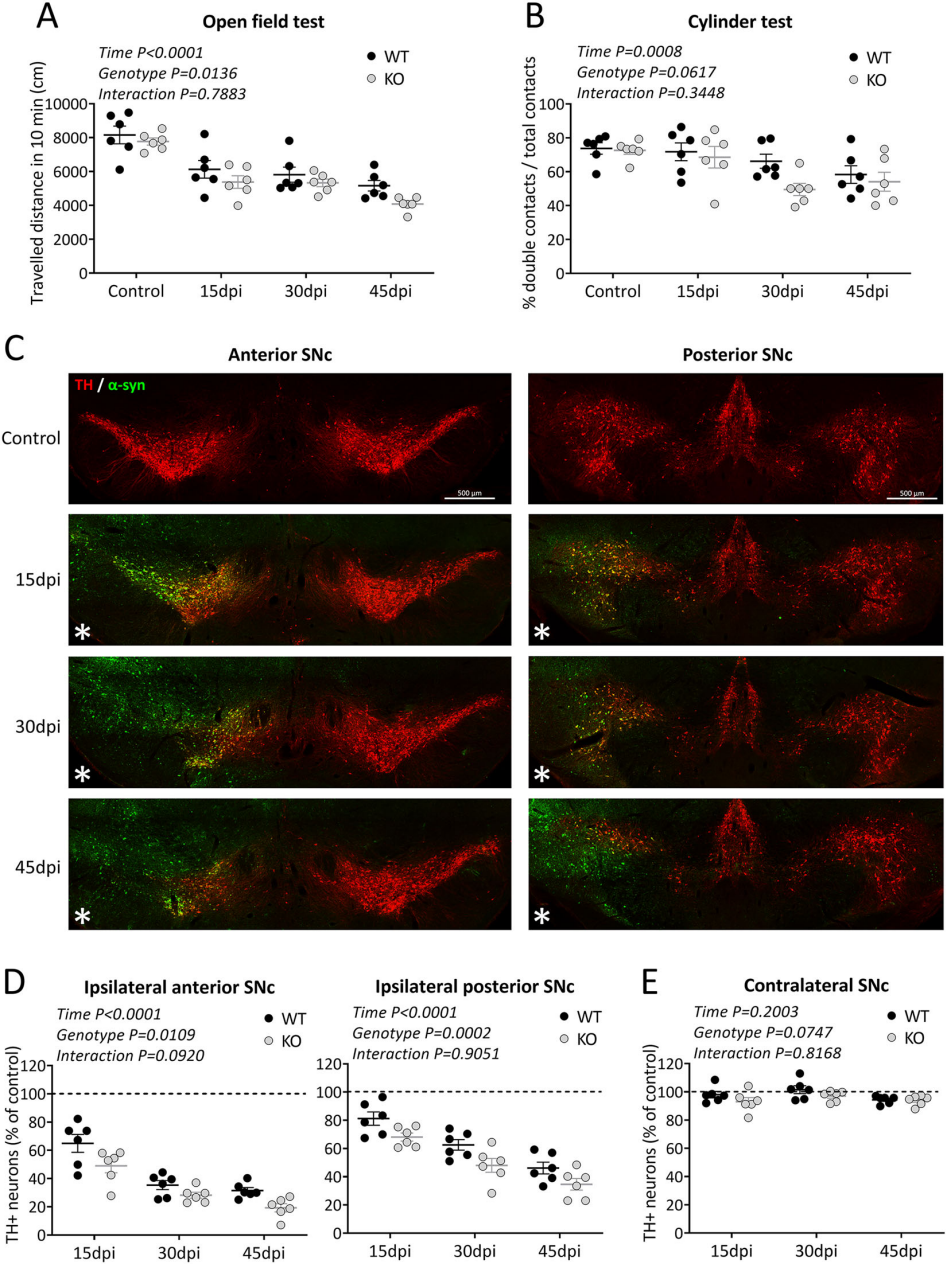
TP53INP1 deficiency aggravates motor deficits and loss of nigral TH-positive neurons induced by AAV-mediated α-synuclein overexpression. **A**, **B** Assessment of motor performance using the open field test for the spontaneous locomotor activity (**A**) and the cylinder test for the parkinsonian-like akinesia (**B**) in WT and *Trp53inp1*^−*/*−^ (KO) control mice and mice with unilateral intranigral injection of AAV2-CBA-human-α-synuclein at 15, 30, and 45 dpi. The injection leads to progressive deficits for the two genotypes, which were significantly worsened in *Trp53inp1*^−*/*−^ mice for locomotor performance in the open field. **C** Illustrative images of dually immunostained sections showing the time course of human α-synuclein expression and of TH neuron loss in the substantia nigra of WT mice. Note that human α-synuclein detection is limited to the injected side and associated with a progressive reduction in the numbers of labeled TH neurons, while no apparent loss is observed in the contralateral side. **D** Total numbers of TH^+^ neurons in the anterior and posterior subdivisions of the ipsilateral SNc measured in WT and KO mice at 15, 30, and 45 dpi and expressed as % of control. The progressive loss of these neurons both in the anterior and the posterior subdivisions of the injected SN was aggravated in KO mice. **E** Lack of changes in the numbers of TH+ neurons in the overall contralateral SNc at the time points examined in WT and KO mice expressed as a percent of control. In all graphs, data are means ± SEM of 6 mice per group, and the results of the two-way ANOVA are indicated within each graph. Only significant interaction is symbolized.

To investigate the potential role of TP53INP1 under PD-related conditions, we used a model based on targeted overexpression of α-synuclein^41^. Unilateral injection of AAV-αsyn in the SNc of 5 month-old WT and *Trp53inp1*^−*/*−^ mice was followed by a time-course study at 15, 30, and 45 days post-injection, assessing motor dysfunction, human-α-synuclein expression, and DA neurodegeneration in the same groups of WT and *Trp53inp1*^−*/*−^ mice. Regarding the behavioral analyses, the two-way ANOVA for the distance traveled in the open field test showed significant time [F(3,40) = 29.2] and genotype [F(1,40) = 6.7] effects, while no age × genotype interaction [F(3,40) = 0.4]. The locomotor deficit was progressive and globally worsened by TP53INP1 deficiency (Fig. 2A). The two way-ANOVA for the number of double contacts in the cylinder test also showed a time effect [F(3,40) = 6.8] but no significant genotype effect [F(1,40) = 3.7; *P* = 0.0617] or time × genotype interaction [F(3,40) = 1.1] (Fig. 2B). Figure 2C illustrates the dual immunolabelling of TH and human-α-synuclein in WT mice at 15, 30, and 45 days post-injection. Efficient transduction was seen in TH^+^ neurons in the whole anteroposterior extent of the injected SNc and part of the VTA as early as 15 dpi. No staining for human α-synuclein was seen in the contralateral SNc at the time points examined. In the same sections, progressive loss of TH neurons could be observed in the AAV-αsyn-injected SNc without visible loss in the contralateral SNc. Regarding counts of nigral TH^+^ neurons in the injected SNc of WT and *Trp53inp1*^−*/*−^ mice, the two-way ANOVA showed significant time and genotype effects while no interaction for both the anterior [F(3,40) = 144.2 and F(1,40) = 7.1, respectively] and the posterior parts [F(3,40) = 67.1 and [F(1,40) = 16.7, respectively]. Therefore, progressive TH^+^ neuron loss occurred in the two subdivisions of SNc and was globally aggravated in *Trp53inp1*^−*/*−^ versus WT mice (Fig. 2D). No significant change in the estimated numbers of TH^+^ neurons was measured in the overall contralateral SNc of WT and *Trp53inp1*^−*/*−^ mice at the time points examined (Fig. 2E). Counts of Nissl-stained cells at 45 dpi showed significant cell loss in the injected SNc versus controls in both the anterior and posterior regions, which was significantly exacerbated in *Trp53inp1*^−*/*−^ compared to WT mice in the anterior SNc (Supplemental Fig. 1B). Again, no change was found in the contralateral SNc (Supplemental Fig. 1C). These data strongly suggest that the ipsilateral decrease in the number of TH^+^ neurons in this model reflects cell death and that neurodegeneration is aggravated under TP53INP1 deficiency.

Together, these results provide evidence for a neuroprotective role of TP53INP1 in PD-like stress conditions.

### Nigral *Trp53inp1* and *Trp53inp2* gene expression in mice is significantly modified in α-synuclein-induced but not ageing-related stress condition

**Fig. 3:**
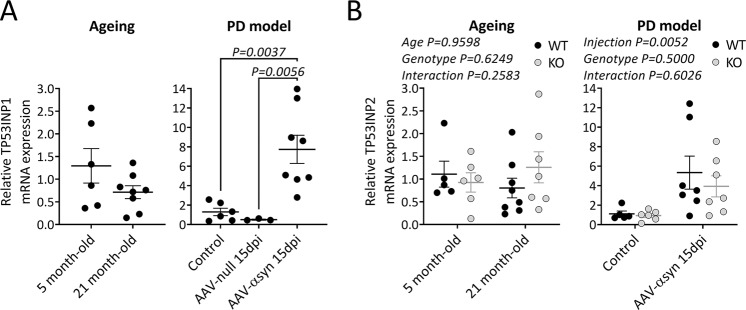
Nigral expression of *Trp53inp1* and *Trp53inp2* increases after AAV-mediated α-synuclein overexpression but not in aged mice. **A** Relative *Trp53inp1* mRNA expression, normalized to *Hprt* housekeeping gene, was measured by RT-qPCR in WT mice at 21 months and at 15 dpi of AAV2-CBA-human-α-synuclein or of AAV null and expressed as fold change versus 5 month-old control mice using the 2^−ΔΔCt^ method. Data are expressed as means ± SEM of *n* mice per group (ageing study: 6 mice of 5 month-old, 8 mice of 21 month-old; PD model: 6 controls, 8 AAV-αsyn and 3 AAV null). *Trp53inp1* expression was not significantly modified with ageing. It was markedly increased in the SN after the injection of AAV2-CBA-human-α-synuclein both versus control and versus AAV null injection, values from the latter group showing no significant difference compared to control; one way ANOVA followed by the Holm–Sidak test. **B** Relative *Trp53inp2* mRNA expression, normalized to *Hprt* housekeeping gene, measured by RT-qPCR in WT and *Trp53inp1*^−*/*−^ (KO) mice at 21 months and at 15 dpi of AAV2-CBA-human-α-synuclein and expressed as fold change versus 5 month-old control mice using the 2^−ΔΔCt^ method. *Trp53inp2* expression was not significantly modified in aged WT and KO mice. In the PD model, it was significantly increased with no genotype difference. Data are expressed as means ± SEM of n mice per group (ageing study: 5 WT and 6 KO of 5 month-old, 8 WT and 7 KO of 21 month-old; PD model: control: 5 WT and 6 KO, AAV-αsyn: 7 WT and 7 KO). Two-way ANOVA results are indicated above each histogram.

Because *TP53INP1* expression has been shown to be enhanced in response to stress in inflamed tissues^1^ and in aged bone marrow^42^, we measured *Trp53inp1* transcript levels in the SN in our experimental conditions, by means of RT-qPCR. *Trp53inp1* mRNA levels did not significantly differ in 21 versus 5 month-old WT mice, although a tendency towards decrease was noticed (−44.9 ± 0.1%) (Fig. 3A). In the PD model, a sixfold increase was found in the SN of WT mice at 15 days post-injection of AAV2-CBA-α-synuclein (*P* = 0.0037 versus control), whereas the levels measured in AAV-null injected animals at the same time point did not differ from control values (Fig. 3A). The values close to zero in *Trp53inp1*^−*/*−^ mice, either uninjected (0.02 ± 0.01, *n* = 6) or injected with AAV-αsyn (0.008 ± 0.010, *n* = 5), confirmed the specificity of the primers used.

We also studied *Trp53inp2*, the paralog of *Trp53inp1*, which is also implicated in autophagy and is induced under cellular stress conditions (Fig. 3B). Nigral *Trp53inp2* transcript levels did not show significant ageing [F(1,22) = 0.003] or genotype-associated change [F(1,22) = 0.3], but were markedly increased at 15 days post-injection of AAV2-CBA-α-synuclein ([F(1,21) = 9.7] both in WT and *Trp53inp1*^−*/*−^ mice, with no difference between the two genotypes [F(1,21) = 0.5]. These data demonstrate that *Trp53inp1* deletion does not affect *Trp53inp2* expression and that expression of the two genes changes in parallel in the conditions examined, being unaffected under ageing and increased in the PD model.

### Neuronal overexpression of *dDOR* is neuroprotective in fly models of PD

**Fig. 4:**
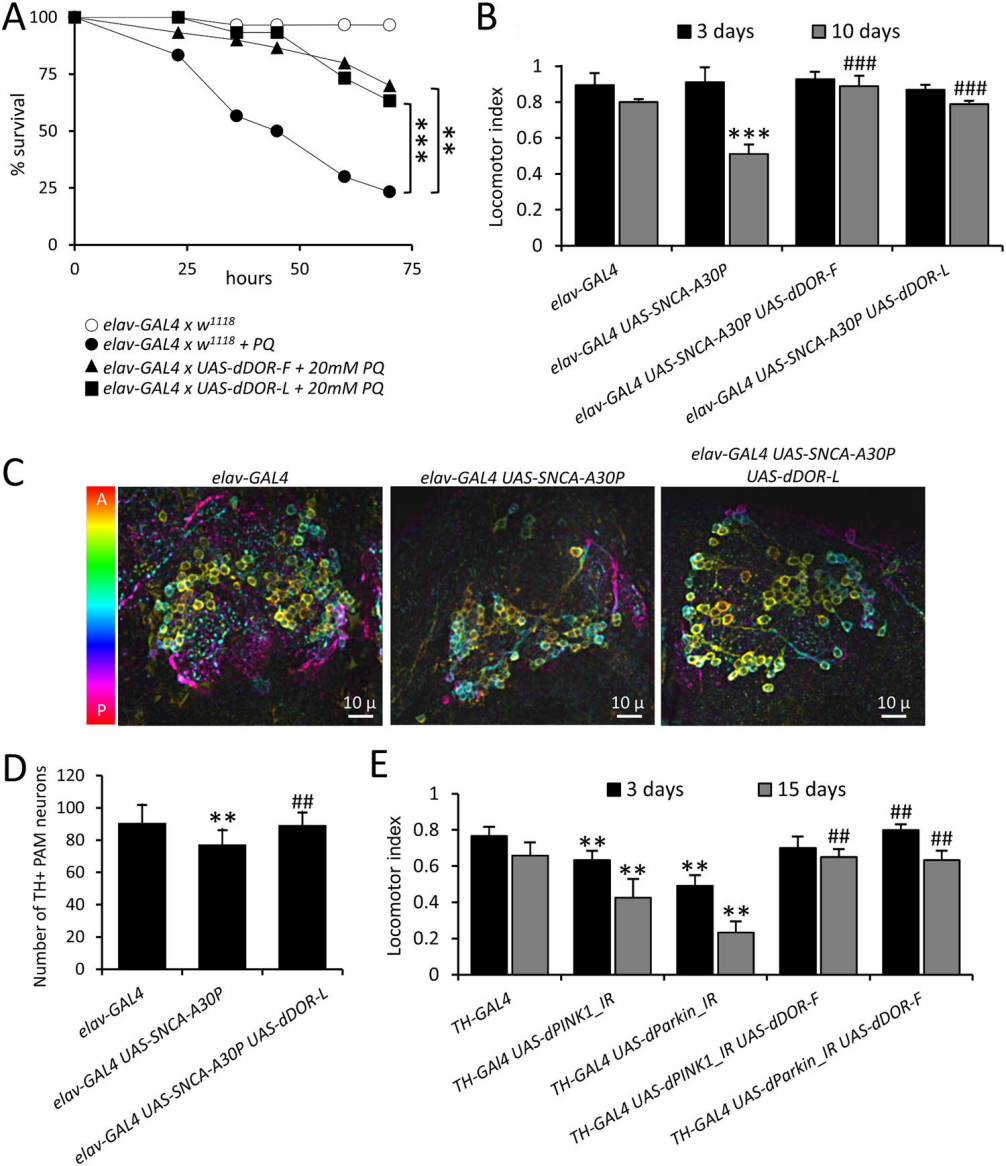
Targeted neuronal overexpression of dDOR is neuroprotective in *Drosophila* models of Parkinson’s disease. **A** Survival curves on a 70-h period, showing the percent of survival of female flies fed with 2% sucrose (white disks) or poisoned with 20 mM Paraquat (PQ) diluted in 2% sucrose (black boxes, disks and triangles). UAS-*dDOR* transgenes (*dDOR-F* but not *dDOR-L* contains EcR binding motif) were overexpressed using a pan-neuronal *GAL4* driver (*elav-GAL4*) and compared to the survival rate of *elav-GAL4* flies crossed to *w*^*1118*^ controls. Survival assay was performed on three groups of ten flies in each condition. Log-rank test results: **P < 0.01, ***P < 0.0001. **B** Locomotor index determined by negative geotaxis tests performed on 3- and 10-day old adult male flies grown at 29 °C. Genotypes analyzed: hemizygous *elav-GAL4* control flies (progeny of *elav-GAL4* females and *w*^*1118*^ males), flies overexpressing dDOR isoforms in neurons (*elav-GAL4 UAS-dDOR-F* and *L*), flies overexpressing human α-synuclein gene p.Ala30Pro in neurons (*elav-GAL4 UAS-SNCA-A30P*), flies overexpressing both dDOR isoforms and human α-synuclein gene p.Ala30Pro in neurons (*elav-GAL4 UAS-SNCA-A30P UAS-dDOR-F* and *L*). Mean of 9 races involving 30 flies in total (±SD). Mann–Whitney *U* test results: *elav-GAL4 UAS-SNCA-A30P* versus *elav-GAL4* control ***P < 0.001; *elav-GAL4 elav-GAL4 UAS-SNCA-A30P* versus *elav-GAL4 UAS-SNCA-A30P UAS-dDOR-F or UAS-dDOR-L ###P* < *0.001*. **C** Z-projections of confocal image stacks showing DA neurons of the protocerebral anterior medial cluster (PAM) after TH immunolabelling of whole mounted brains from 15 days old adult male flies. Genotypes analyzed: hemizygous *elav-GAL4* control males (progeny of *elav-GAL4* females and *w*^*1118*^ males), flies overexpressing human α-synuclein gene p.Ala30Pro in neurons (*elav-GAL4 UAS-SNCA-A30P*), flies overexpressing dDOR-L and human α-synuclein gene p.Ala30Pro in neurons (*elav-GAL4 UAS-SNCA-A30P UAS-dDOR-L*). The color code represents the Z-depth of the stack on the anterior (A)–posterior (P) brain axis. **D** Quantification of TH-positive neurons in PAM clusters imaged in brains of 15 days old adult male flies. Number of clusters analyzed in each genotype: 15 for *elav-GAL4*, 16 for *elav-GAL4 UAS-SNCA-A30*, 15 for *elav-GAL4 UAS-SNCA-A30P UAS-dDOR-L*. Mann–Whitney *U* test results: *elav-GAL4 UAS-SNCA-A30P* versus *elav-GAL4* control **P < 0.01; *elav-GAL4 elav-GAL4 UAS-SNCA-A30P* versus *elav-GAL4 UAS-SNCA-A30P UAS-dDOR-L ##P* < *0.01*. **E** Locomotor index determined by negative geotaxis tests performed on 3- and 15-day old adult male flies grown at 29 °C. Genotypes analyzed: heterozygous *TH-GAL4* control flies (progeny of *TH-GAL4* females and *w*^*1118*^ males), flies overexpressing RNAi against *Drosophila dPINK1* or *dParkin* in DA neurons (*elav-GAL4 UAS-dPINK1_IR or UAS-dParkin_IR*), and flies overexpressing both dDOR-F and RNAi against *dPINK1* or *dParkin* in DA neurons (*elav-GAL4 UAS-dPINK1_IR or UAS-dParkin_IR UAS-dDOR-F*). All genetic conditions include a *UAS-Dicer2* transgene. Mean of 9 races involving 30 flies in total (±SD). Mann–Whitney *U* test results: *TH-GAL4 UAS-dPINK1_IR* or *UAS-dParkin_IR* versus *TH-GAL4* control **P < 0.01; *TH-GAL4 UAS-dPINK1_IR* or *UAS-dParkin_IR* versus *TH-GAL4 UAS-dPINK1_IR* or *UAS-dParkin_IR UAS-dDOR-F ##P* < *0.01*.

To explore further the neuroprotective role of TP53INPs highlighted by the above findings, we characterized the consequence of modulating the expression of *dDOR*, the *Drosophila* homolog of *TP53INP1/TP53INP2*, in fly models of PD. We first used a model based on the exposure of adult flies to the mitochondrial stressor and neuronal toxin paraquat (PQ), which decreases their lifespan. The specific over-expression of splicing variants *dDOR-F* or *dDOR-L*, encoding respectively protein isoforms with or without the ecdysone receptor (EcR) binding motif^32^, improved the resistance of adult flies to PQ, as revealed by a partial recovery in the survival assay (Fig. 4A). Then, we overexpressed *dDOR* in fly models mimicking hereditary forms of PD. Negative geotaxis test and TH immunostaining revealed that neuronal *dDOR* overexpression rescued the progressive locomotor decline and the DA neuron loss in the PAM cluster observed in flies overexpressing the p.Arg30Pro allele of human *SNCA* in neurons (Fig. 4B–D). *dDOR* overexpression specifically targeted to TH-expressing neurons also restored locomotion in flies with RNAi-induced downregulation of either *dPINK1* or *dParkin* in these neurons (Fig. 4E). Taken together, these results show that *dDOR* over-expression in neurons provides neuroprotection in flies exposed to toxins or to genetic alterations linked to PD.

### dDOR activates autophagy and basal mitophagy in fly neurons

**Fig. 5:**
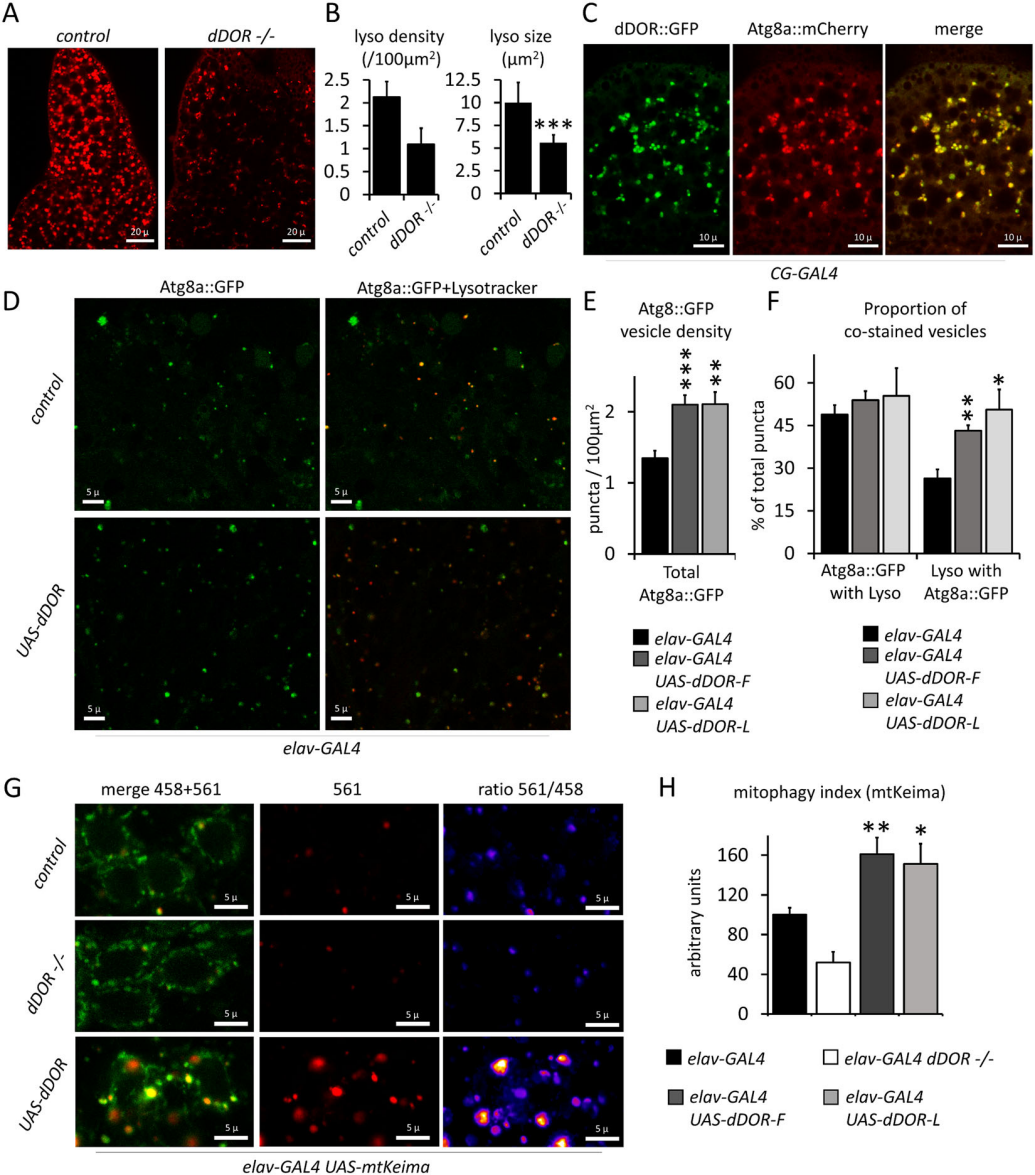
*TP53INP1Drosophila* homolog *dDOR* activates autophagy and mitophagy. **A** Confocal images of living third instar larvae fat bodies from control (*w*^*1118*^) and *dDOR* homozygous null mutant (−/−) stained with Lysotracker. **B** Quantification of lysosome density (lysotracker positive puncta per 100 µm^2^) (left panel) and quantification of average lysosomal size (µm ± SD) in control and *dDOR* homozygous mutant fat bodies. Three samples analyzed for each condition. Mann–Whitney *U* test result: ***P < 0.001. **C** Confocal images of living fat bodies from *CG-GAL4 UAS-mCherry::Atg8a UAS-dDOR::GFP* third instar larvae. **D** Confocal images of *elav-GAL4 UAS-Atg8a::GFP* living adult fly brains (10-day old females at 29 °C) stained with lysotracker and expressing or not a *UAS-dDOR-F* transgene. **E** Quantification of Atg8a::GFP positive vesicles (puncta per 100 µm^2^ ± SEM) in brains of *elav-GAL4 UAS-Atga::GFP* adult flies expressing or not a *UAS-dDOR* transgenes (dDOR-F but not dDOR-L contains EcR binding motif). Number of analyzed samples: 20 for *elav-GAL4* control, 14 for *elav-GAL4 UAS-dDOR-F*, 8 for *elav-GAL4 UAS-dDOR-L*. Mann–Whitney *U* test results: ***P < 0.001, **P < 0.001. **F** Proportion of Atg8::GFP puncta co-stained with Lysotracker (% ±SEM) and proportion of Lysotracker positive puncta co-stained with Atg8a::GFP in flies expressing or not *UAS-dDOR* transgenes. Number of analyzed samples: 20 for *elav-GAL4* control, 14 for *elav-GAL4 UAS-dDOR-F*, 8 for *elav-GAL4 UAS-dDOR-L*. Mann–Whitney *U* test results: **P < 0.01, *P < 0.05. **G** Confocal images of *elav-GAL4 UAS-mtKeima* living adult brains (7-day old females at 29 °C) from control, *dDOR* homozygous mutant (−/−) or *UAS-dDOR* transgenic flies. Left panels show a superposition of mtKeima signals captured upon 458 nm (green chanel) and 561 nm (red channel) excitation. Central panels show mtKeima signal upon 561 nm excitation only. Right panels show ratiometric images obtained by dividing mtKeima signal intensities upon excitation at 561 nm and 458 nm. **H** Quantification of mitophagy index (±SEM) measured in *elav-GAL4 UAS-mtKeima* living adult brains from control, *dDOR* homozygous mutant or *UAS-dDOR-F and dDOR-F* transgenic flies. Mitophagy index corresponds to total pixel intensity of ratiometric images (mtKeima signal at 561 nm/signal at 458 nm) normalized by total mitochondrial area. Number of analyzed samples: 15 for *elav-GAL4* control, 9 for *elav-GAL4 dDOR*^−/−^ 9 for *elav-GAL4 UAS-dDOR-F*, 7 for *elav-GAL4 UAS-dDOR-L*. Mann–Whitney *U* test result: **P < 0.01.

We next explored potential mechanisms underlying the neuroprotective role of *dDOR* in flies. RNAi approaches previously suggested that *dDOR* is a positive regulator of the developmentally-regulated autophagy that occurs in fat bodies in *Drosophila* larvae^43^. We confirmed that dDOR regulates autophagy in fat bodies, as shown by the strong decrease in the density and size of lysotracker-positive puncta in *dDOR* null mutant larvae (Fig. 5A, B) and by the colocalization of dDOR::GFP fusion protein with Atg8a-positive autophagolysosomes (Fig. 5C). We then investigated the role of dDOR in neurons by overexpressing *dDOR-F* or *dDOR-L* splicing variants. Both isoforms similarly increased the density of Atg8a positive puncta in the fly brain (Fig. 5D, E), suggesting that dDOR promotes autophagy in neurons independently of the ecdysone pathway. Although the number of Atg8a::GFP vesicles increased upon dDOR overexpression, the proportion of those that co-stained with lysotracker was similar to that of control (Fig. 5F). In contrast, the proportion of lysosomes that co-stained with Atg8::GFP was increased, indicating that lysosomes are increasingly participating in autophagy. Taken together, these results demonstrate that *dDOR* overexpression fosters the maturation of autophagosomes into autophagolysosomes. Because it was recently suggested that TP53INP1 promotes mitophagy in a mouse model of metabolic syndrome^8^, we quantified basal mitophagy levels in *Drosophila* neurons in which *dDOR* was either inactivated or upregulated, using the dual excitation ratiometric fluorescent probe mtKeima^33^. The 561 nm/458 nm signal ratio, which reflects mitochondria delivered to the acidic lysosomal compartment, was decreased in *dDOR* knockout (KO) neurons and increased in neurons overexpressing *dDOR* (Fig. 5G, H). These findings show that dDOR acts as a positive regulator of basal mitophagy in neurons, a process minimally affected by dPINK1 or dParkin deficiency^33^.

### *dDOR* does not genetically interact with *dPINK1*

**Fig. 6:**
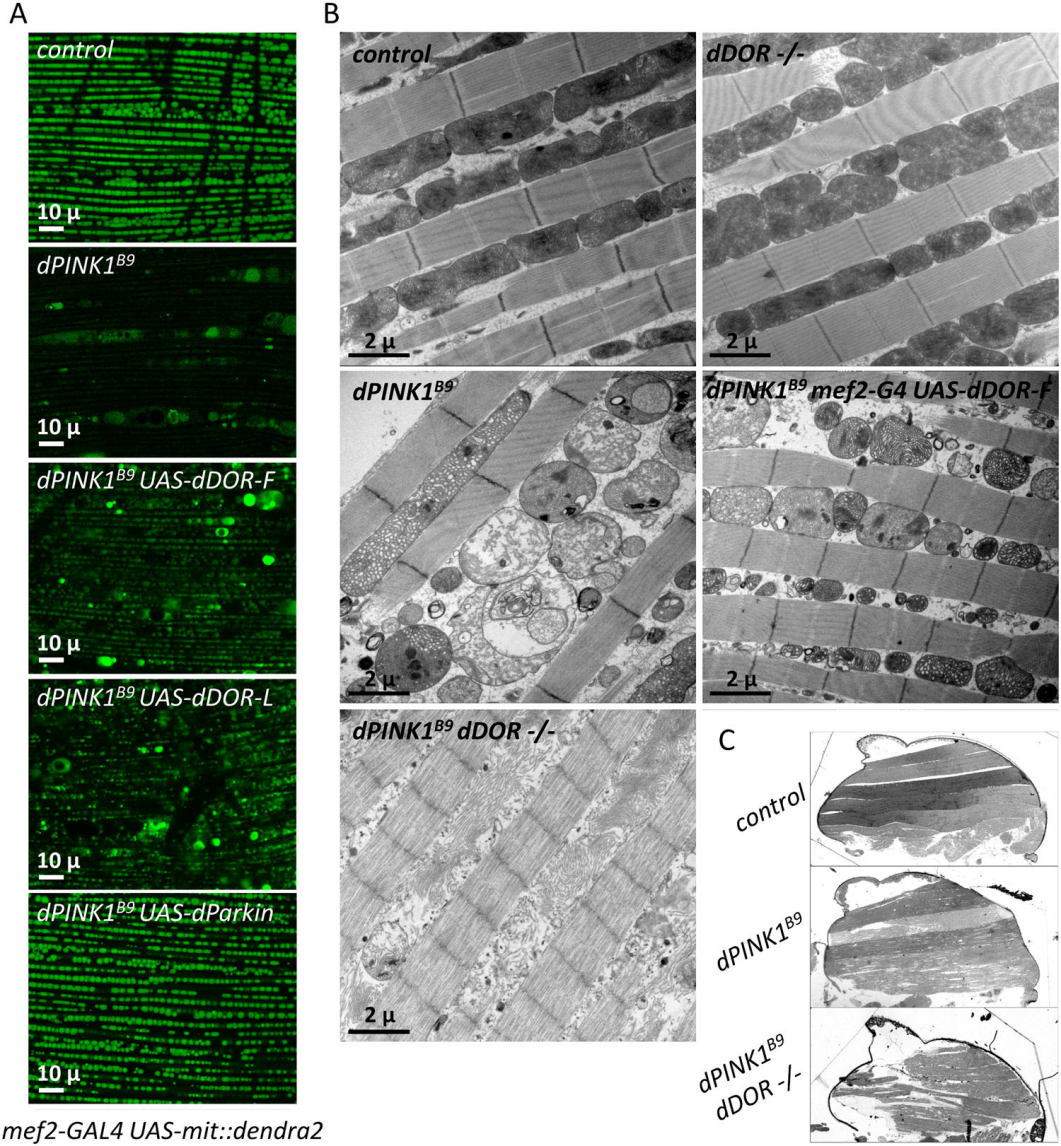
*dDOR* overexpression rescues while its downregulation aggravates *dPINK1* mutant phenotype in drosophila flight muscles. **A** Living *mef2-GAL4 UAS-mit::dendra2* (staining of the mitochondrial matrix) adult male dorsal longitudinal flight muscles (DLM) from control, *dPINK1*^*B9*^ hemizygous mutant or *dPINK1*^*B9*^ hemizygous mutant expressing *UAS-dDOR* transgenes (*dDOR-F* but not *dDOR-L* contains EcR binding motif). **B** Transmission electron microscopy images of adult fly thorax sections showing mitochondrial ultrastructure in DLM muscle of control, homozygous *dDOR* null mutant, *dPINK1*^*B9*^ hemizygous mutant, *dPINK1*^*B9*^ hemizygous mutant expressing *UAS-dDOR-F* and *dPINK1*^*B9*^ hemizygous mutant combined with homozygous *dDOR* null mutation. **C** Fly thorax sections of control, *dPINK1* hemizygous mutant and *dPINK1* hemizygous mutant combined with *dDOR* homozygous mutation stained with Toluidine blue showing DLM muscles. In dPINK1 hemizygous mutant, DLM muscles contain intact fibers that span longitudinally, as seen in the control fly. In dPINK1 hemizygous mutant combined with dDOR homozygous mutation, the extremities of DLM muscle appear ripped off from the cuticle and the muscle fibers are disorganized and torn. Four thoraces were analyzed for each condition.

We explored the potential interplay between *dDOR* and the mitophagy-associated gene *dPINK1* using the canonical model of dorsal longitudinal indirect adult flight muscles (DLM) that has been used to highlight the genetic interactions between *dPINK1* and *dParkin*^44,45^. *dDOR-F or dDOR-L* overexpression partially rescued the mitochondrial alterations triggered by *dPINK1* inactivation, including the reduced mitochondrial matrix fluorescent staining (Fig. 6A), the loss of mitochondrial membrane integrity (Fig. 6B), and the presence of abnormally enlarged mitochondria (Fig. 6A, B). Of note, *dDOR* was however less efficient than *dParkin* in rescuing the *dPINK1* mutant phenotypes (Fig. 6A). *dDOR* mutant flies did not exhibit any obvious phenotype in DLM. In contrast to previous data showing that *dPINK1*; *dParkin* double mutants are phenotypically similar to the respective single mutants^46^, the *dPINK1*; *dDOR* double mutants exhibited a more severe degenerative phenotype in DLM than the single *dPINK1* mutant, at both microscopic and macroscopic levels (Fig. 6B, C). In these flies, only the remains of mitochondrial membranes were observable (Fig. 6B). In addition, muscle fibers were strongly affected (Fig. 6B), leading to flight muscle degeneration and disorganization visible at the macroscopic scale (Fig. 6C). Altogether, these data show that *dDOR* regulates basal mitophagy in parallel to *dPINK1* and *dParkin*, possibly by activating autophagy.

### TP53INP1 promotes autophagy but does not affect PINK1/Parkin-dependent mitophagy in mammalian cells

Others and we have previously reported a role of TP53INP1 in starvation-induced autophagy^5,36^. Here, we investigated whether TP53INP1 also promotes autophagy in COS7 cells (Supplemental Fig. S2). To this end, we co-expressed a TP53INP1-YFP fusion protein with the autophagosomal marker LC3 fused to the fluorescent protein dsRed (LC3-DsRed) and determined its effect on basal and starvation-induced autophagy. To do so, we incubated the cells in normal medium or in medium deprived of all amino acids, in the presence or absence of bafilomycin A1, a common inhibitor of autophagosome-lysosome fusion and autolysosome acidification. In cells incubated with normal medium and not treated with bafilomycin A1 (control), LC3-positive vesicles covered on average less than 0.5% of the total cell area (relative LC3-positive area), independently of TP53INP1 overexpression. Treatment with bafilomycin A1 led to a sevenfold increase in the relative LC3-positive area in non-starved cells and a 25-fold increase in starved cells with endogenous TP53INP1 level compared to control condition (Supplemental Fig. S2A, B). Overexpression of TP53INP1 exacerbated these effects, leading to an 11-fold increase in the relative LC3-positive area in non-starved cells and a 34-fold increase in starved cells. Furthermore, the intracellular distribution of TP53INP1-YFP in the different conditions fitted with the expected mainly nuclear localization of TP53INP1 under basal conditions (–bafilomycin A1), and its recruitment to LC3-DsRed punctate structures following treatment of the cells with bafilomycin A1 (Supplemental Fig. S2A). Taken together, these results confirm the previously reported role of TP53INP1 in the positive regulation of autophagy.

**Fig. 7:**
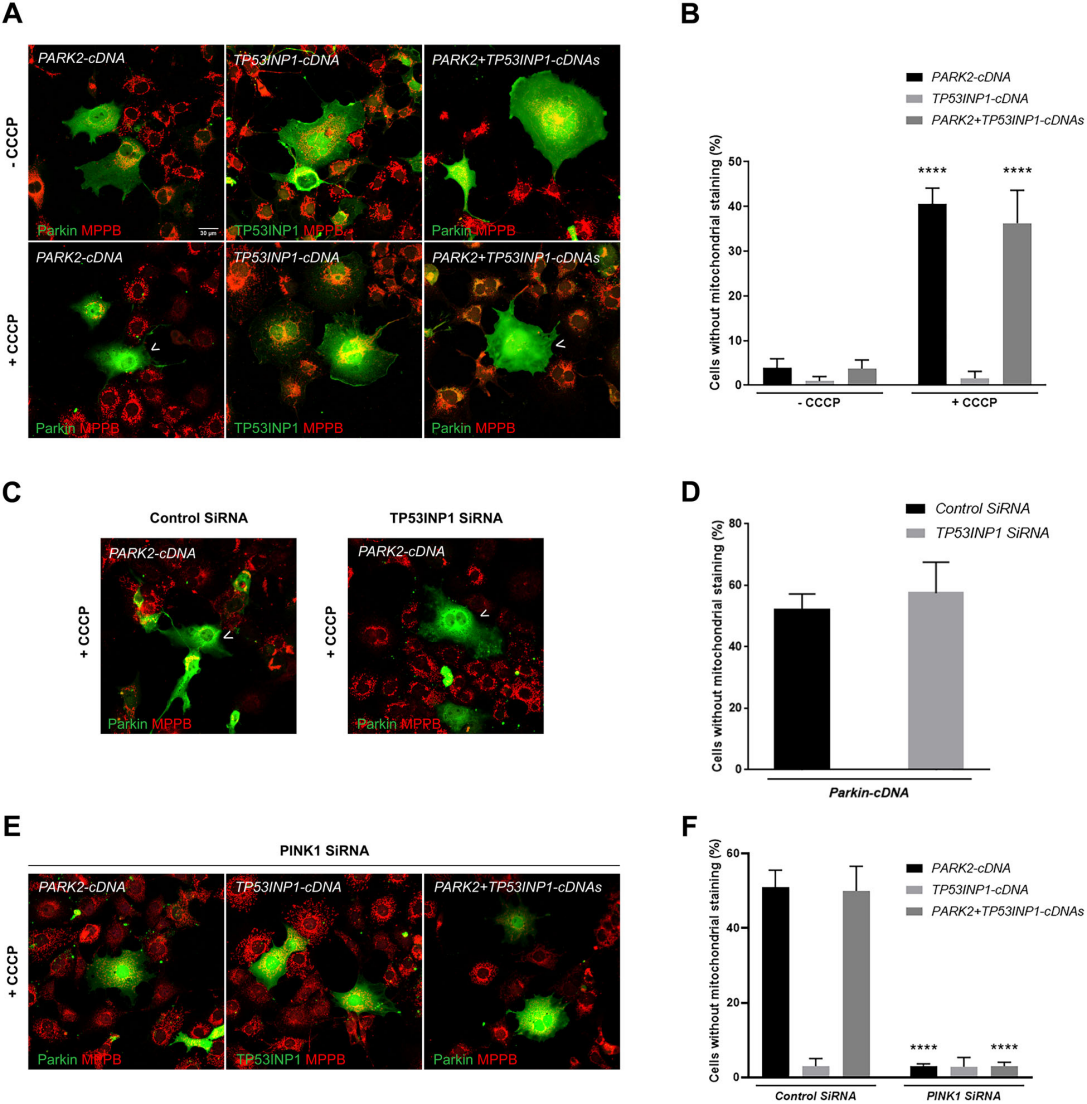
TP53INP1 does not affect PINK1/Parkin-dependent mitophagy in a mammalian cell model. **A** Representative Z-stack projections illustrating clearance of the mitochondrial matrix marker MPPB in COS7 cells overexpressing Parkin alone or with TP53INP1, following CCCP treatment. An arrowhead indicates cells with no or few mitochondria. Note that mitochondria are not cleared in cells overexpressing TP53INP1 only, neither in the presence nor in absence of CCCP. **B** Quantitative analysis of the data presented in (**A**), illustrating the proportion of Parkin-(PARK2-cDNA and PARK2 + TP53INP1-cDNAs) or TP53INP1-positive cells (TP53INP1-cDNA) with no or little MPPB staining in the indicated conditions. ****P < 0.0001 versus respective -CCCP condition (two-way ANOVA followed by Tukey’s test). **C**, **D** Representative Z-stack projections and corresponding quantitative analysis showing mitochondrial (MPPB) clearance triggered by CCCP in cells overexpressing Parkin, silenced or not for *TP53INP1*. *P* < 0.05 (two-tailed *t*-test). **E**, **F** Representative Z-stack projections and corresponding quantitative analysis showing impairment of CCCP-induced Parkin-dependent mitochondrial (MPPB) clearance following *PINK1* silencing, both in the presence (PARK2 + TP53INP1-cDNAs) and absence of TP53INP1 (PARK2-cDNA), and presence of mitochondria in cells overexpressing TP53INP1 and silenced for *PINK1* (TP53INP1-cDNA). ****P < 0.0001 versus respective Control SiRNA condition (two-way ANOVA followed by Tukey’s test). Results are means ± SD, with *n* = 3 independent experiments. Arrowheads indicate cells with virtually no mitochondria.

Our results in *Drosophila* also suggest that TP53INP1/dDOR acts by enhancing autophagy in general rather than specifically promoting PINK1/Parkin-dependent mitophagy. We further tested this hypothesis by investigating the impact of TP53INP1 overexpression or depletion in a mammalian cell model allowing to assess specifically mitochondrial stress-induced PINK1/Parkin-dependent mitophagy (Fig. 7)^37^. COS7 cells, in which endogenous Parkin is undetectable, were transfected to overexpress human Parkin alone or with TP53INP1 and treated with the mitochondrial uncoupling agent carbonyl cyanide chlorophenylhydrazone (CCCP). We verified that most of the cells expressing Parkin also expressed TP53INP1 in the cotransfection condition: 90.5% ± 2.1 of the Parkin-positive cells were also positive for TP53INP1 in absence of CCCP and 78.5% ± 4.6 in its presence (at least 300 cells scored from 3 independent wells per condition). We then investigated mitochondrial clearance by quantifying the proportion of Parkin-positive cells lacking the beta subunit of the mitochondrial processing peptidase (MPPB), a mitochondrial matrix marker, 48 h after CCCP treatment. As expected, about 40% of the Parkin-positive cells had lost their mitochondrial network at this time point (Fig. 7A, B). This proportion was similar in cells co-expressing TP53INP1. In addition, mitochondrial clearance was not observed in cells overexpressing TP53INP1 only. We also examined the effect of siRNA-induced *TP53INP1* silencing on mitochondrial clearance triggered by Parkin. *TP53INP1* transcript levels were reduced to 5.67 ± 0.58% of those measured in cells treated with a control siRNA, as estimated by RT-qPCR using three independent primer pairs. Consistent with our observations in cells overexpressing TP53INP1, downregulation of the endogenous *TP53INP1* gene did not affect the proportion of cells without mitochondria following CCCP treatment (Fig. 7C, D). Finally, we evaluated whether TP53INP1 would be able to trigger mitochondrial stress-induced mitophagy in cells with a dysfunctional PINK1/Parkin pathway. Mitochondrial clearance was quantified in cells expressing TP53INP1 alone or with Parkin and simultaneously silenced for *PINK1* (Fig. 7E, F). Parkin-dependent mitophagy triggered by CCCP was blocked in the absence of PINK1, as expected. This mitophagy defect was not rescued by TP53INP1, whether expressed alone or together with Parkin. Altogether, these results exclude an involvement of TP53INP1 in the regulation of mitochondrial stress-induced PINK1/Parkin-dependent mitophagy.

## Discussion

TP53INP1 has been mainly described as involved in mediating apoptotic cell death, including neuronal death, via activation of p53 signaling. However, a study showing that TP53INP1-deficient mouse thymocytes and embryonic fibroblasts have enhanced stress-induced apoptosis, associated with impaired ROS regulation, defective antioxidant defense, and reduced autophagy, emphasized a role of TP53INP1 in protection against cell injury, possibly through autophagy regulation^47^. Several studies have since stressed the action of TP53INP1 as a regulator of autophagy in non-neuronal cells^5,36^. The present findings in mouse, *Drosophila*, and a mammalian cell model provide the first evidence for the implication of stress-induced proteins of the TP53INP1/DOR family in protecting neurons against age- and ND-related death, as well as in regulating autophagy and basal mitophagy in neurons.

We first addressed the neuroprotection issue in the mouse, by examining the impact of TP53INP1 deficiency on the evolution of midbrain DA neuron numbers with ageing and in a PD model. Our data in WT mice show that the ageing-related loss of TH neurons does not affect the VTA and is restricted in the SNc to the anterior subdivision, which agrees with previous analysis in the rat^48^. We also found that *Trp53inp1* mRNA levels in SN tend to decrease in aged mice, which could reflect an impaired response to stress, possibly related to age-dependent autophagy decline^18,19^. Interestingly, TP53INP1 deficiency does not aggravate the ageing-related loss of TH neurons in the anterior SNc but results in an additional neuronal loss in the posterior SNc. As a possible explanation, the normal ageing-related neurodegeneration may be linked to a regionalized decline of TP53INP1 and of neuronal autophagy in the anterior region, restricting the deleterious effect of TP53INP1 deficiency to the caudal region. Therefore, TP53INP1 expression may protect neurons of the caudal SNc against ageing-related neurodegeneration. Regarding PD, DA neuron fallout and associated pathological changes have been reported to be prominent in the ventrolateral and caudal SN^13,49,50^. The PD model based on targeted overexpression of α-synuclein in the SN does not reproduce this pattern, cell loss evolving more rapidly and predominantly in the anterior subdivision of the SNc. Nevertheless, the results obtained argue for an implication of TP53INP1 in cell defense against PD-related stress in this model: nigral *Trp53inp1* expression increases at an early stage of degeneration, possibly due to the neuroinflammation occurring in this model^51^; consistent with a pro-survival role of this response, TP53INP1 deficiency exacerbates motor deficits and loss of nigral TH neurons. Altogether, the data obtained in mice suggest that TP53INP1 exerts neuroprotection under ageing and PD-related stress condition. Since the studies were conducted exclusively in male mice, one cannot exclude sex differences in the role of this stress-induced protein.

In the two stress conditions examined, TP53INP1 deficiency induced moderate additional loss of DA neurons. Whether TP53INP2 can offset TP53INP1 deficiency in neuroprotective mechanisms is an important issue. The two proteins have different transcriptional activity: TP53INP1 transactivates p53 and p73, underlying its potent pro-apoptotic action, while TP53INP2 coactivates nuclear hormone receptors^5^ and has been recently implicated in death receptor-mediated apoptosis^52^. In contrast, TP53INP1 and TP53INP2 share an LC3-interacting region (LIR) and act as positive regulators of autophagy^5,36,47,53^. TP53INP2 binds to the LC3/GABARAP/Atg8 proteins, as does TP53INP1, but also to the autophagosome transmembrane proteins VMP1 and Atg7, presumably acting through this interaction as a scaffold protein that recruits Atg proteins to the autophagosome membrane^53,54^. We show here that TP53INP2 mRNA levels are not modified by TP53INP1 deficiency, indicating a lack of compensation at the gene expression level. However, this does not exclude redundant/complementary functions in the maintenance of neuronal homeostasis via autophagy regulation in our experimental conditions. Notably, as for *Trp53inp1*, gene expression of *Trp53inp2* is increased in the PD model, and this overexpression is maintained in the *Trp53inp1*^*−/−*^ mouse. In this respect, *Drosophila* is an excellent model system because there is only one *TP53INP1/2* homolog in the fly genome, *dDOR*.

As for its mammalian homologs TP53INP1 and 2, dDOR is a dual regulator of autophagy and transcription. It has been described as an Atg8a/b interactor regulating autophagy in *Drosophila* larval fat bodies^32,43^, as confirmed in this study. dDOR is also a coactivator of nuclear hormone receptor (like TP53INP2), which potentiates ecdysone hormone signaling during larval development^32^. Of note, these two molecular activities may have a converging impact since ecdysone signaling induces programmed autophagy in the fat body^55^. The role of *dDOR* in the context of NDs has never been investigated. To address this issue and further discriminate between the two activities of *dDOR*, we compared the effects of overexpressing *dDOR-F* or *dDOR-L* in different *Drosophila* PD models. Whereas both dDOR-F and dDOR-L sequences share an LIR domain, dDOR-F additionally contains an EcR-binding motif (FENLL) contributing to signaling of the molting hormone ecdysone^32^. Pan-neuronal expression of either splice variant reduced the deleterious effect of paraquat on fly survival and alleviated the locomotor defect and the loss of DA neurons exhibited by flies overexpressing *SNCA*^*A30P*^. The EcR binding motif is thus dispensable for the neuroprotection provided by *dDOR*, which argues in favor of action via autophagy regulation. Moreover, *dDOR* overexpression in DA neurons rescued the locomotor decline associated with *dPINK1* or *dParkin* RNAi-mediated dowregulation in these neurons, suggesting that *dDOR* compensates for the function of these PD-related genes.

To provide insights into the putative mechanism underlying *dDOR-*mediated neuroprotection, we investigated the interplay between *dDOR* and the autophagic and mitophagic processes in fly neurons in vivo by examining the effects of *dDOR-L* and *F* overexpression via a pan-neuronal driver. We demonstrate that *dDOR* positively regulates autophagy in adult fly neurons, enhancing the production of autophagosomes and their maturation into autophagolysosomes, independently of the ecdysone signaling. It has been reported that TP53INP1-deficiency impairs mitophagy in non-neuronal cells and proposed that TP53INP1 could either act as a protein cargo directing mitochondria for degradation to autophagosomes or contribute to PINK/Parkin-dependent mitophagy^8^. Here, we show that dDOR is a positive regulator of mitophagy in fly neurons, *dDOR* KO and overexpression resulting respectively in decreased and increased mitophagy. By epistasis analysis in the canonical model system of indirect adult flight muscles^44,45^, we further provide evidence that *dDOR* acts on mitophagy in parallel of the PINK1-pathway: *dDOR* overexpression partially rescues the phenotype associated with *dPINK1* downregulation whereas its deficiency aggravates the *dPINK1* mutant phenotype. Interestingly, another regulator of autophagy, Atg1, rescues mitochondrial alterations in the muscles of *dPINK1* and *dParkin* mutants^56^. Taken together, these observations suggest that dDOR acts as a modulator of mitophagy level by regulating autophagic flux rather than through the PINK1-mediated mitophagy pathway. In this connection, dPINK1 and dParkin have been reported to have minimal impact on basal mitophagy in vivo under physiological conditions^33^, while they may be crucial in mitophagy induction in vivo under ageing or mitochondrial stress triggered by toxin or hypoxia^57,58^. Finally, we show that neither overexpressing nor downregulating *TP53INP1* affects mitochondrial stress-induced PINK1/Parkin-dependent mitophagy in a mammalian cell model classically used to study this process^37,59^. In addition, *TP53INP1* overexpression is unable to restore mitophagy following *PINK1* silencing, a condition well known to abolish Parkin-dependent mitochondrial clearance. These results provide evidence against the implication of TP53INP1 in mitochondrial stress-induced PINK1/Parkin-mediated mitophagy in mammalian cells, supporting the view that dDOR/TP53INP1 and PINK1 may promote mitophagy through two independent pathways.

Taken together, our data uncover an unsuspected role of TP53INP1/dDOR in neuroprotection under ageing and PD-related conditions. Whether the neuroprotection evidenced in the mouse models is cell-autonomous or non-cell-autonomous remains to be determined. For instance, single-cell RNA-seq expression profiles show that *Trp53inp1* is expressed in various cell types, including neurons, glial and endothelial cells, with region-specific differences in the mouse brain^60^. How these profiles are modified under stress conditions is an open and interesting issue. In addition, the study in *Drosophila* identifies dDOR as a novel regulator of basal mitophagy in neurons likely via autophagy regulation. The neuroprotective effects observed here in a diversity of models associated with mitochondrial dysfunction or abnormal protein accumulation suggest that TP53INP1/dDOR might adapt basal autophagy and mitophagy demands to chronic stress conditions requiring efficient turnover of unfolded proteins and damaged organelles to sustain neuronal homeostasis. This pathway may help neurons to face ageing- and NDs-related alterations in parallel to a homeostatic mechanisms involving PINK1 and Parkin.

## Supplementary information

Supplemental figure Legend

Supplemental Figure S1

Supplemental Figure S2

## Data Availability

The data generated during this study are included in this publication.
