# Supplementary material for: TP53INP1 exerts neuroprotection under ageing and Parkinson’s disease-related stress condition
Source: Cell Death Dis. 2021 May 8;12(5):460. doi: 10.1038/s41419-021-03742-4 (PMC8106680; doi:10.1038/s41419-021-03742-4)
Supplement: Supplementary file 1 — Supplemental figure Legend [file 41419_2021_3742_MOESM1_ESM.docx]

**Supplemental Figure Legend**

**Figure S1. TP53INP1 deficiency induces additional loss of nigral Nissl-stained neurons under ageing and AAV-mediated α-synuclein overexpression.**

A. Ageing-related evolution of Nissl-stained neuron numbers in the anterior and posterior SNc of WT and *Trp53inp1^-/-^* (KO) mice. Counts were performed in 5 and 21 month-old animals of the two genotypes and results expressed as % of 5 month-old WT mice. In the anterior SNc, WT and KO mice show equivalent numbers of Nissl-stained neurons at the two ages considered and equivalent neuron loss at the age of 21 months vs 5 months. In the posterior SNc, the number of cells is significantly reduced in KO vs WT 21 month-old mice. B. Estimated numbers of Nissl-stained neurons in the anterior and posterior subdivisions of the AAV α-syn-injected SNc measured in WT and KO mice at 45 dpi and expressed as % of control. KO mice show aggravated loss of these neurons versus WT in the anterior SN. C. Estimated numbers of Nissl-stained neurons in the whole contralateral SNc of WT and KO mice at 45 dpi expressed as % of control. No significant changes are measured. In all graphs, data are expressed as means ± S.E.M. of n mice per group (ageing study: 6 WT and 6 KO of 5 month-old, 8 WT and 8 KO of 21 month-old; PD model: 6 control and 6 AAV α-syn at 45 dpi per genotype) and results of the two-way ANOVA are indicated. Only significant genotype difference is symbolized.

**Figure S2. TP53INP1 promotes autophagy in mammalian cells.**

A. Representative Z-stack projections illustrating LC3-DsRed positive autophagic vesicles in starved or non-starved cells with endogenous TP53INP1 levels (-TP53INP1-cDNA) or overexpressing a TP53INP1-YFP fusion protein, following treatment or not with the lysosome inhibitor bafilomycin A1. Note the recruitment of TP53INP1 to LC3-positive vesicles in cells treated with bafilomycin A1. **B**. Quantitative analysis of the fold increase in relative LC3-positive area versus control (non-starved, - bafilomycin A1, - TP53INP1-cDNA) in the different conditions presented in A. Results are means ± SEM, with n = 25-32 cells per condition from one experiment representative of two. The two-way ANOVA shows significant starvation effect (P<0.0001), transfection effect (P<0.01) and no significant interaction between starvation and transfection condition. Significant transfection effect is indicated **P<0.01.
