# Supplementary figures and images for: TP53INP1 exerts neuroprotection under ageing and Parkinson’s disease-related stress condition
Source: Cell Death Dis. 2021 May 8;12(5):460. doi: 10.1038/s41419-021-03742-4 (PMC8106680; doi:10.1038/s41419-021-03742-4)

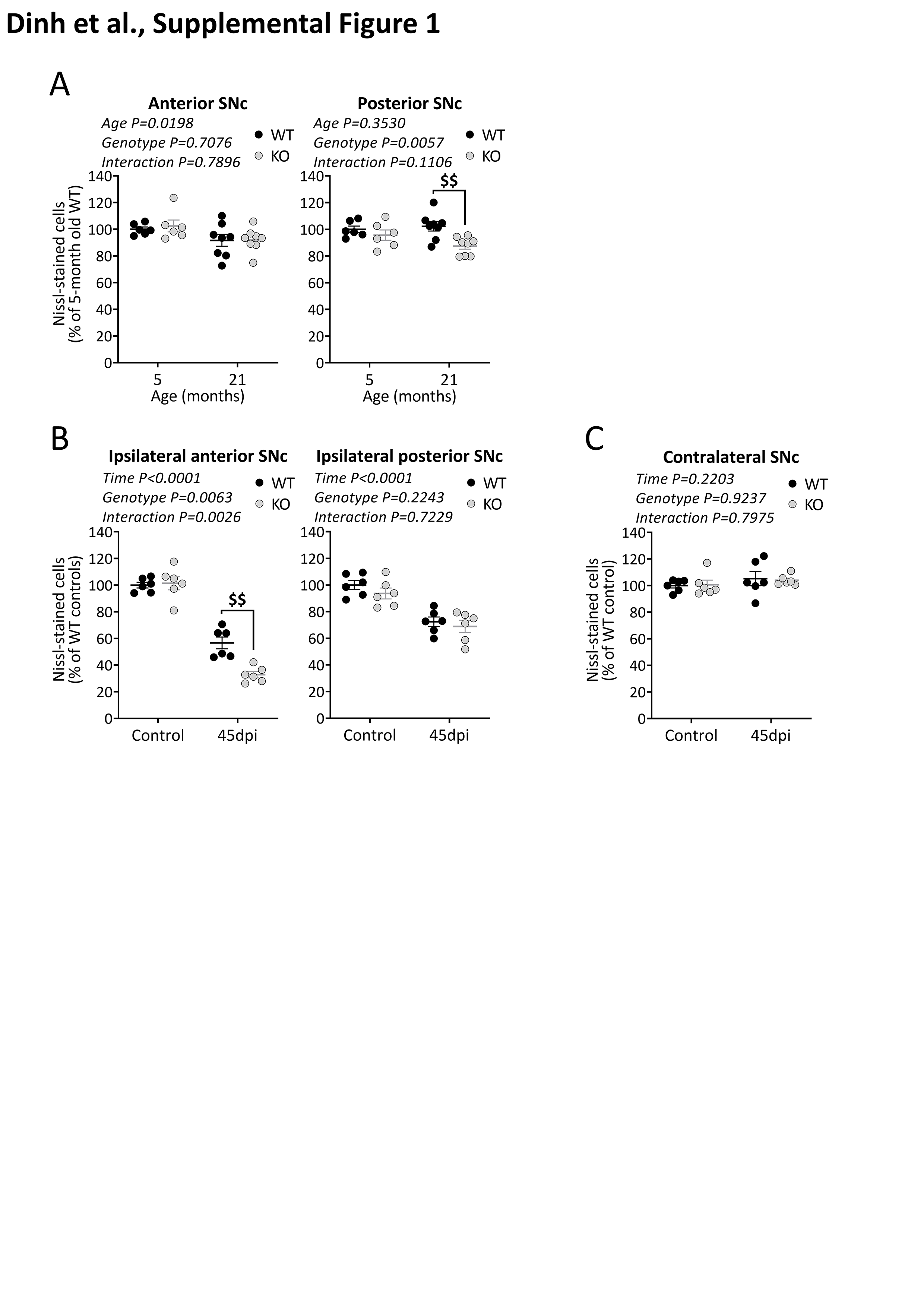

Supplement: Supplementary file 2 — Supplemental Figure S1 [file 41419_2021_3742_MOESM2_ESM.tif]

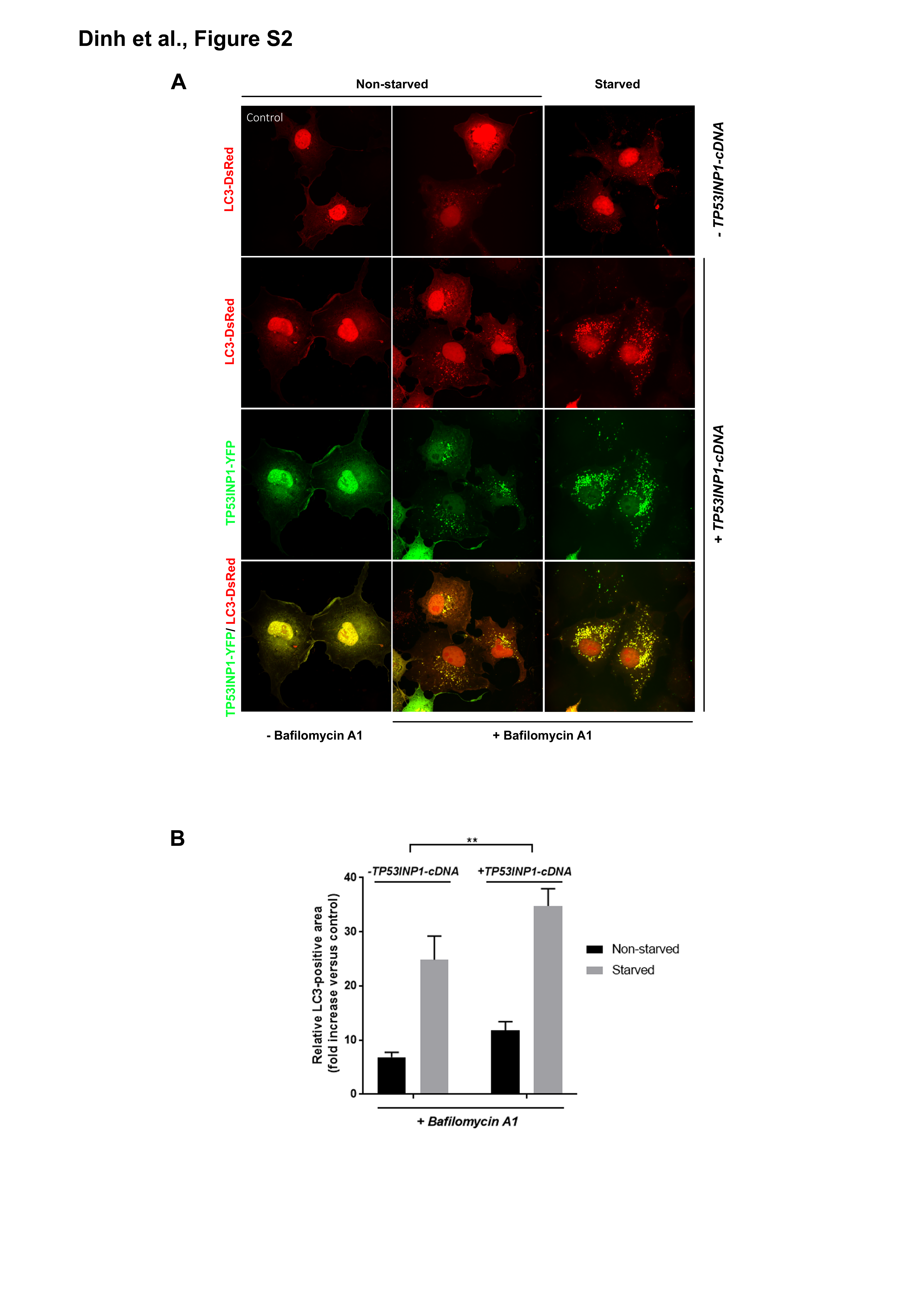

Supplement: Supplementary file 3 — Supplemental Figure S2 [file 41419_2021_3742_MOESM3_ESM.tif]
